# SpyCEP dismantles neutrophil immunity via disorder-driven chemokine remodeling and GAG targeting

**DOI:** 10.1073/pnas.2520164123

**Published:** 2026-07-09

**Authors:** Rikin J. Lau, Sean P. Giblin, Andra Sugar, Antonio Di Maio, Giulio Tassini, Kristin Huse, Dror Chorev, Yuan Chen, Grace Ho-Yan Wu, Camilla Berg Huemer, Seung Yon Kim, Jayden Matthews, Bel Muloud, Lu Chen, Sophie McKenna, Yingqi Xu, Luisa Massai, Chiara Muzzi, Xhenti Ferhati, Francesca Necchi, Danilo Gomes Moriel, Ten Feizi, Yan Liu, James E. Pease, Shiranee Sriskandan, Steve Matthews

**Affiliations:** ^a^ Department of Life Sciences, Imperial College London, London SW7 2AZ, United Kingdom; ^b^ Centre for Bacterial Resistance Biology, Imperial College London, London SW7 2AZ, United Kingdom; ^c^ National Heart and Lung Institute, Imperial College London, London W12 0NN, United Kingdom; ^d^ Glycosciences Laboratory, Department of Metabolism, Digestion and Reproduction, Faculty of Medicine, Imperial College London, London W12 0NN, United Kingdom; ^e^GlaxoSmithKline Vaccines Institute for Global Health, Siena 53100, Italy; ^f^ Department of Infectious Disease, Imperial College London, London W12 0NN, United Kingdom; ^g^CTM Technologies and Materials Ltd., Ness-Ziona 7403626, Israel

**Keywords:** *Streptococcus pyogenes*, SpyCEP, CXCL8, cryo-EM, NMR

## Abstract

*Streptococcus pyogenes* (Group A *Streptococcus*) employs sophisticated virulence strategies to evade human immunity, including secretion of the cell envelope protease SpyCEP, which cleaves and inactivates key neutrophil-attracting chemokines such as CXCL8. Here, we integrate cryo-electron microscopy, NMR spectroscopy, and native mass spectrometry to investigate how SpyCEP disrupts CXCL8 function. We demonstrate that a disordered aromatic and acidic region within the cleaved autocatalytic maturation loop (CAML) of SpyCEP mimics receptor N-domains and binds an allosteric site on CXCL8. The resulting interaction forms a dynamic fuzzy complex and is coupled to dimer dissociation, consistent with enhanced access to the cleavage site. This disorder-mediated substrate engagement differs from classical protease mechanisms that rely on rigid recognition interfaces. Additionally, glycan microarray and NMR analyses show that the CAML region mediates glycosaminoglycan (GAG) binding, suggesting a means for SpyCEP to maximize encounters with GAG-enriched CXCL8 reservoirs. Together, these findings provide a structural and biophysical framework for understanding how SpyCEP combines substrate engagement with GAG targeting to dismantle chemokine gradients and inhibit neutrophil recruitment. More broadly, this work highlights the role of intrinsic disorder in protease recognition and suggests avenues for anti-virulence therapies and vaccine strategies targeting SpyCEP.

## Significance

*Streptococcus pyogenes* evades neutrophil-mediated immunity by secreting the protease SpyCEP, which inactivates chemokines such as CXCL8; however, the mechanism by which SpyCEP targets CXCL8 for cleavage has remained unclear. This work uncovers an intrinsically disordered autocatalytic maturation loop that binds CXCL8 and induces a conformationally heterogeneous state in the chemokine. A model is proposed in which this disorder-mediated recognition facilitates access to the substrate cleavage site and is compatible with SpyCEP acting at glycosaminoglycan (GAG)-bound CXCL8 reservoirs. This disorder-mediated mode of substrate recognition departs from classical protease–substrate interfaces and identifies the SpyCEP cleaved autocatalytic matu-ration loop (CAML) as a potential target for anti-virulence strategies against *S. pyogenes*.

*Streptococcus pyogenes* (Group A *Streptococcus*; GAS) has evolved an exquisite adaptation to its human host manifesting in a range of different diseases, such as mild cases of pharyngitis or impetigo, invasive toxic shock syndrome and necrotizing fasciitis, and immune sequelae leading to rheumatic heart disease (1). *S. pyogenes* is one of the leading causes of infection-related deaths worldwide. Despite its significant medical burden and being a priority in the WHO’s Global Vaccine Action Plan, no vaccine exists for *S. pyogenes* (2, 3). Among its virulence factors, *S. pyogenes* secretes SpyCEP, a protease that disrupts immune responses by cleaving the entire family of CXC chemokines possessing N-terminal “ELR” motifs, most notably interleukin 8 (CXCL8 or IL8) (4–7). SpyCEP is an excellent vaccine candidate because antibodies against it block protease activity and confer protection in invasive models (8, 9).

CXCL8, a central mediator of neutrophil-driven inflammation, exerts its function through interactions with both G protein–coupled receptors (CXCR1/CXCR2) and glycosaminoglycans (GAGs) (10). CXCL8 interacts with GAGs predominantly through its basic C-terminal helix with contributions from the N-loop regions (11–13), which serves as a scaffold for chemokine dimerization and presentation, ensuring the establishment of productive chemotactic gradients (14). Furthermore, the GAG interaction offers protection from proteolytic cleavage by the stabilization of dimeric chemokine structures and concealment of cleavage sites. SpyCEP removes a large portion of the GAG-binding helix from CXCL8, thereby preventing chemokine recruitment to GAGs and activation of neutrophil receptors (5, 15, 16).

SpyCEP faces a formidable structural challenge, as it targets a site at the heart of the chemokine tertiary structure that contributes to dimer stabilization (17) and sequestration within GAG networks (18). Unlike other proteases that target accessible chemokine termini, SpyCEP must significantly remodel its substrate to proceed with enzymatic cleavage, and in doing so neutralizes both soluble and surface-tethered pools of chemokines. Here, we combine single particle cryoelectron microscopy (EM), NMR, and native mass spectrometry (MS) to unveil a multistep mechanism to facilitate CXCL8 cleavage, combining substrate destabilization, monomer sequestration, and self-recruitment to GAGs. We demonstrate that an intrinsically disordered region (IDR) of the cleaved autocatalytic maturation loop (CAML) not only targets SpyCEP to the GAG-bound CXCL8 reservoir but also induces a conformationally heterogeneous state in CXCL8 that, with the assistance of the protease-associated (PA) domain, facilitates monomer capture and access to the buried cleavage site. This disorder-driven unfolding represents a significant departure from classical protease mechanisms, which typically rely on an ordered binding interface to recognize preaccessible sites. Although IDRs are increasingly recognized as critical elements in protein–protein interactions, their role in driving substrate remodeling had not been demonstrated until now.

The broader implications of this work are twofold. First, it highlights the underappreciated role of intrinsic disorder in proteases and challenges the view that they rely solely on rigid, structured interfaces for substrate engagement. Second, it provides a structural framework for understanding how *S. pyogenes* subverts neutrophil-driven immunity, offering potential avenues for antivirulence therapies and vaccine design.

## Results

### Single Particle Cryoelectron Microscopy Analyses of SpyCEP–CXCL8 Complexes.

Despite significant effort, there has been no structural description of SpyCEP in complex with its bound substrate. To address this fundamental gap in our understanding of such a crucial virulence enzyme, we embarked on a joint cryoelectron microscopy (cryoEM) and NMR study of the structural dynamics of SpyCEP and its interaction with substrate CXCL8. Previously, only the crystal structures of mature SpyCEP in the absence of substrate have been solved (19, 20). Intriguingly, cleavage of a 70 aa autocatalytic maturation loop (AML) generates two halves (Cleaved Autocatalytic Maturation Loop, CAML comprising CAML_NT_ and CAML_CT_), which are presumed flexible as they were not observed in crystal structures (19, 20). Furthermore, the PA domain was not well-resolved in the electron density and could not be fully traced.

**Fig. 1.**
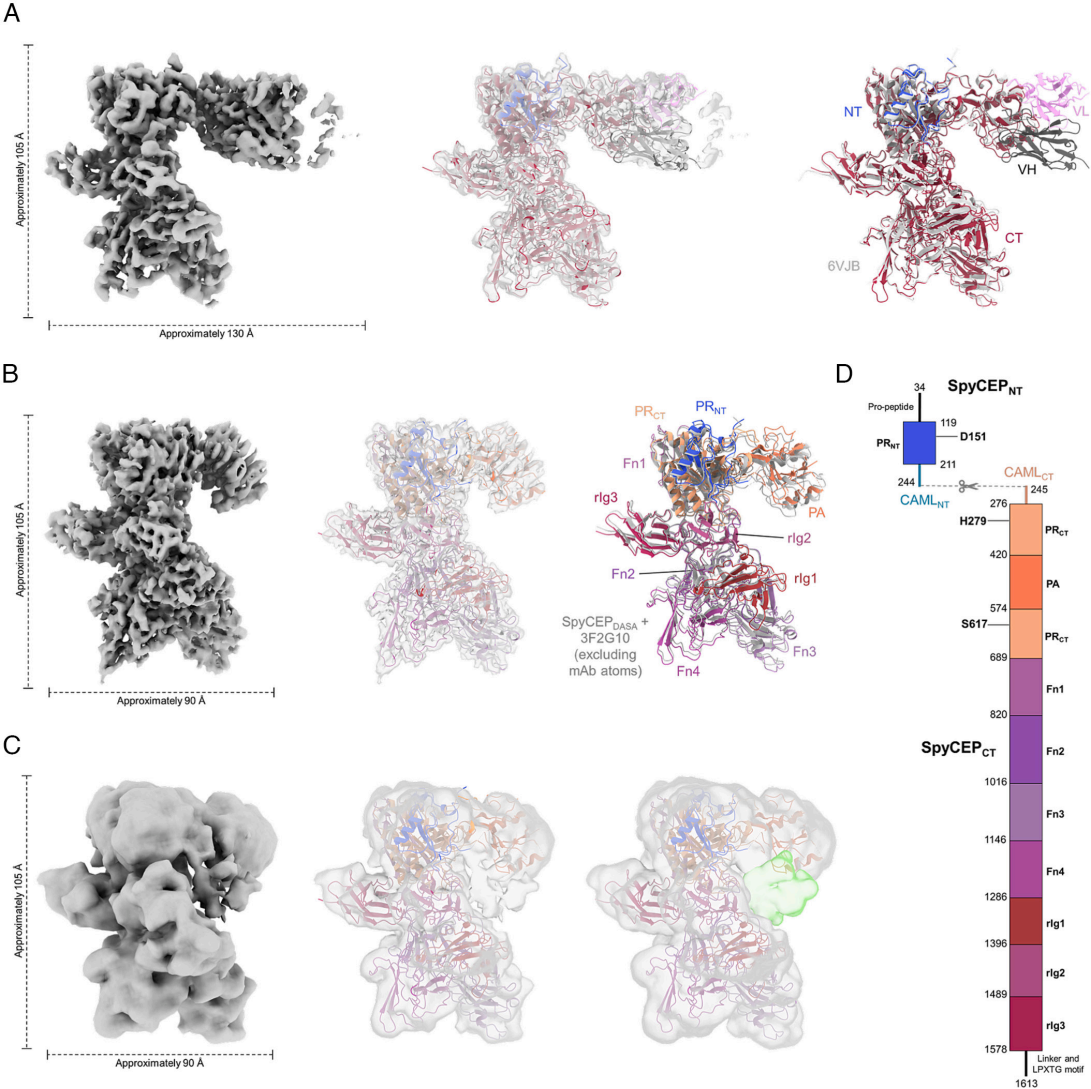
Single particle cryoelectron microscopy analyses of SpyCEP–CXCL8 complexes. (*A*) Cryo-EM 3D reconstruction of the SpyCEP–antibody complex after refinement (*Left*: 3.07 Å) with ISOLDE fitted crystal structure (*Middle*) and cartoon representation of the superimposition with crystal structure of SpyCEP alone (*Right*; pdb:6VJB). (*B*) Cryo-EM 3D reconstruction of the SpyCEP–CXCL8 complex (*Left*: 3.61 Å) with ISOLDE fitted structure (*Middle*) and cartoon representation of the superimposition with the SpyCEP structure from *A*. (*C*) Cryo-EM 3D reconstruction of the crosslinked SpyCEP–CXCL8 complex (*Left*: 6.65 Å) with rigid body fitted SpyCEP structure (*Middle*) and CXCL8 density highlighted in green. (*D*) Domain organization of the mature SpyCEP heterodimer (SpyCEP_NT and_ SpyCEP_CT_). Color coded the same in the structures showing in *A*–*C*.

To provide a molecular scaffold for improved alignment in cryoEM single particle analysis, we prepared gel-filtered complexes of the SpyCEP_D151A, S617A_ (SpyCEP_DASA_) mutant, which retains structure and immunogenicity but is proteolytically inactive (19), with two noninhibitory monoclonal antibodies (3F2G10 and 10B6C10, *SI Appendix*, Fig. S1*A*). While the 10B6C10 antibody bound to a disordered linear epitope at the N terminus of SpyCEP (*SI Appendix*, Figs. S1*B*, S2, and S3) and did not result in an enhanced map, 3F2G10 bound to a conformational epitope on the distal side of the PA domain and facilitated a significant improvement in 3D reconstructions of SpyCEP (3.07 Å, 660 k particles; Fig. 1*A* and *SI Appendix*, Figs. S2 and S3 and Table S1). The SpyCEP N-terminal protease subunit and protease-associated (PA) domains are extended with four fibronectin (Fn) and three reverse-immunoglobulin (rIg) domains, which coil back and contact the protease domain (Fig. 1*B*). In this closed structure the active site sits at the base of a large crater delineated by the protease, PA and Fn2-4 domains, and is inaccessible to a folded CXCL8 dimer. Despite being located near the active site, the CAML was not represented in any 3D reconstructions, which confirms the significant conformational disorder. The PA domain, however, could now be fully traced and this model was used to interpret EM reconstructions with bound CXCL8 (Fig. 1*A*).

The interaction between SpyCEP_DASA_ and CXCL8 is of high affinity (21), therefore we chose to mix enzyme with substrate and immediately apply the complex to a cryo-EM grid. Processing produced a 3D reconstruction of a SpyCEP heterodimer (3.61 Å, 96 k particles; Fig. 1*B* and *SI Appendix*, Figs. S4 and S5 and Table S1), but the dataset lacked obvious evidence for a bound substrate suggesting the interaction was not retained during grid preparation or that bound CXCL8 is extremely dynamic and averaged. An overlay of the reconstruction with our complete SpyCEP model generated from the antibody complex, showed no additional density corresponding to CXCL8. After refinement of the model within the map (22), the whole SpyCEP structure appeared more open with the PA domain moving away by ~3.7 Å, which may indicate the presence of a bound CXCL8 that is dynamic.

As SpyCEP may exist in a dynamic complex or form a transient CXCL8 interaction state, we attempted to capture the complex using covalent cross-linking with formaldehyde (23). SDS-PAGE confirmed crosslinking of CXCL8 with SpyCEP_DASA_ (*SI Appendix*, Fig. S4), and the purified fraction was applied to cryo-EM grids. After rounds of enrichment, a distinct class was identified as a SpyCEP–CXCL8 complex as it showed additional electron density representing CXCL8. After training with Topaz, 739 k particles were processed down to 105 k, and three 3D classes were generated (Fig. 1*C* and *SI Appendix*, Figs. S4 and S5). Class 1 exhibited the best density for CXCL8-bound SpyCEP, representing 35 k particles at 6.35 Å. Although class 0 contained CXCL8 density, the PA domain was not intact; class 2 lacked CXCL8.

After 3D refinement of class 1, the electron density of CXCL8 was visible at lower contour, suggesting significant conformational heterogeneity and poor averaging of flexible structural ensembles (Fig. 1*C*). Comparison of class 1 of the CXCL8-linked model with the unbound SpyCEP model showed that the SpyCEP enzyme adopts a very similar conformation with additional density positioned between the active site and the PA domain that is consistent with a CXCL8 monomer. We hypothesized that the flexible CAML and PA domain mediate CXCL8 binding, due to their proximity to the CXCL8 density and inherent flexibility explaining why the interaction could only be captured after crosslinking.

### Identification of the CXCL8 Binding Interface within SpyCEP.

**Fig. 2.**
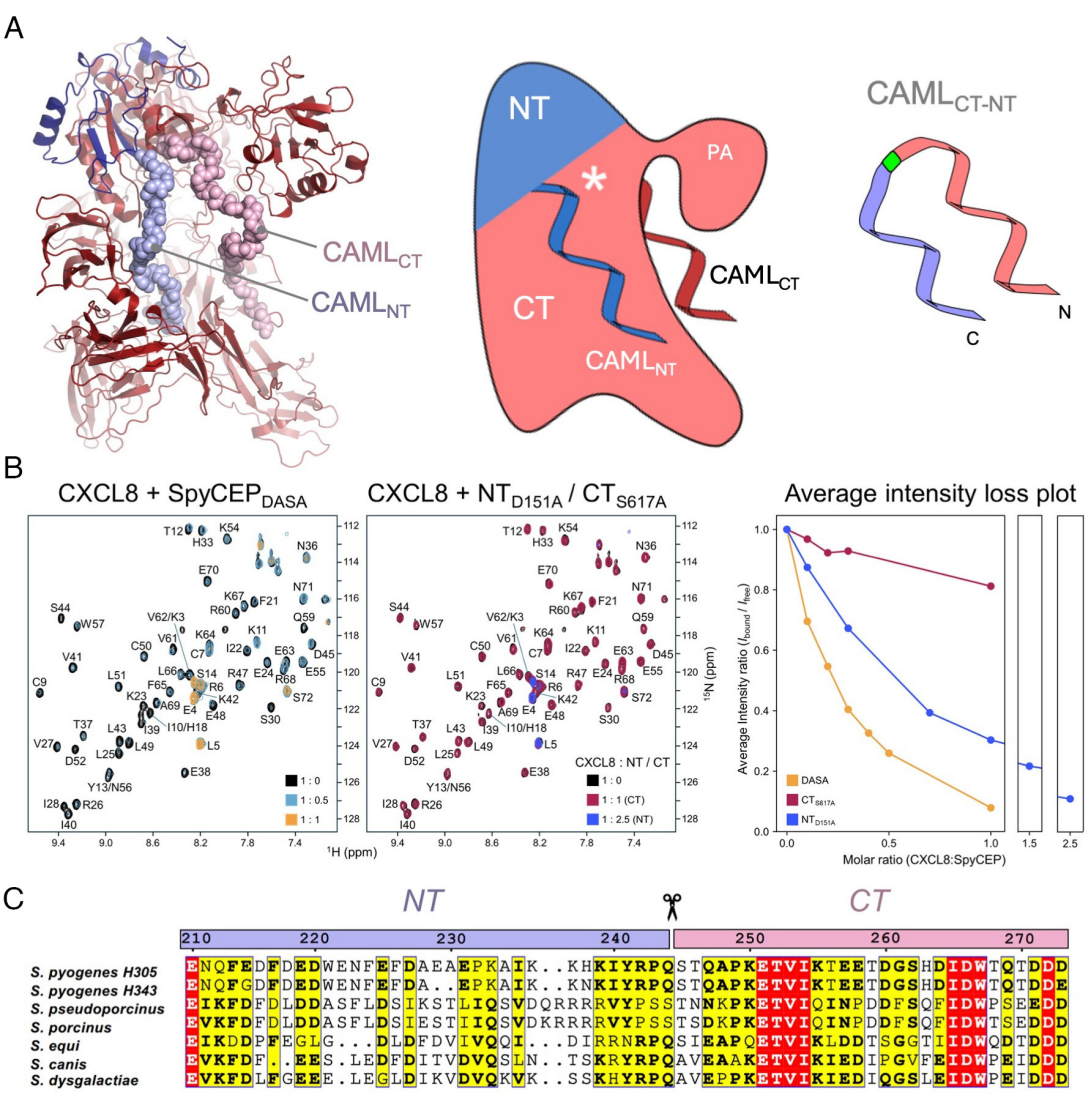
Identification of the CXCL8 binding interface within SpyCEP. (*A*) Cartoon and schematic representation of the SpyCEP heterodimer structure with a modeled CAML to show its juxtaposition to the main body of the enzyme (*Left* and *Middle*). Schematic representation of the cleaved autocatalytic maturation loop (CAML_CT-NT_) construct used for NMR spectroscopy characterization. SpyCEP_NT_ in red/pink and SpyCEP_CT_ in blue/light blue. (*B*) ^1^H-^15^N HSQC NMR titrations of ^15^N labeled CXCL8 with full-length SpyCEP heterodimer (*Left*), SpyCEP_NT_ and SpyCEP_CT_ individually (*Middle*), and plot of intensities versus molar ratio of titrant (*Right*). (*C*) Sequence alignment of representative SpyCEP CAMLs highlighting conserved features.

The conformational heterogeneity observed in the EM reconstruction of the SpyCEP–CXCL8 complex and their proximity, raised the notion that CXCL8 may interact with the flexible CAML within SpyCEP (SpyCEP_CAML_; Fig. 2*A*). To provide further clues into the SpyCEP–CXCL8 interaction interface we performed a series of NMR titrations. We first monitored the ^1^H-^13^C methyl TROSY spectrum of ILV-labeled full-length SpyCEP_DASA_ in the presence of CXCL8 to confirm binding and estimate the dissociation constant. The interaction was in slow exchange on the NMR timescale with a calculated K_D_ of ~500 nM (*SI Appendix*, Fig. S6*A*). Upon titration of unlabeled SpyCEP_DASA_ into ^15^N-labeled CXCL8, almost all resonances in the ^1^H-^15^N HSQC spectrum broadened and disappeared at a 1:1 molar ratio (Fig. 2*B*). The broadening was generally consistent across the spectrum, with most peaks experiencing intensity reductions at low molar ratios and disappearing by the end of the titration, while peaks assigned to the signature ELR motif of CXCL8 exhibited narrow linewidths at the end of the titration. The global peak attenuation could be explained by the formation of a rigid 180+ kDa complex, for which spin–spin relaxation would be very fast, or a heterogeneous ensemble exhibiting conformational exchange.

To determine which of these possibilities is most likely, we next titrated ^15^N-labeled CXCL8 with either of the isolated N- or C-terminal chains of SpyCEP (SpyCEP_NT_ D151A and SpyCEP_CT_ S617A) and recorded their NMR spectra. Unexpectedly, the larger 151 kDa SpyCEP_CT_, which retains enzyme structure (*SI Appendix*, Fig. S6*B*) and activity (21), showed no significant spectral perturbations with CXCL8 (Fig. 2*B*). This raised the notion that the N-terminal fragment recognized CXCL8. Consistent with this, CXCL8 titrated with the 24 kDa SpyCEP_NT_ showed the same spectral behavior as with the full SpyCEP_DASA_ heterodimer, with the entire spectrum disappearing except for the N-terminal ELR motif. With a total Mw <40 kDa, the complex should be NMR visible, so the severe resonance broadening must be due to conformational heterogeneity within the complex. As the majority of the SpyCEP_NT_ sequence adopts a folded structure upon incorporation into the native enzyme, we postulated that the major CXCL8-interacting region lies within the disordered SpyCEP_CAML_ of SpyCEP_NT_, which contains a striking acidic and aromatic-rich region (Fig. 2*C* and *SI Appendix*, Fig. S6*C*).

### SpyCEP CAML Induces Conformational Heterogeneity in CXCL8.

**Fig. 3.**
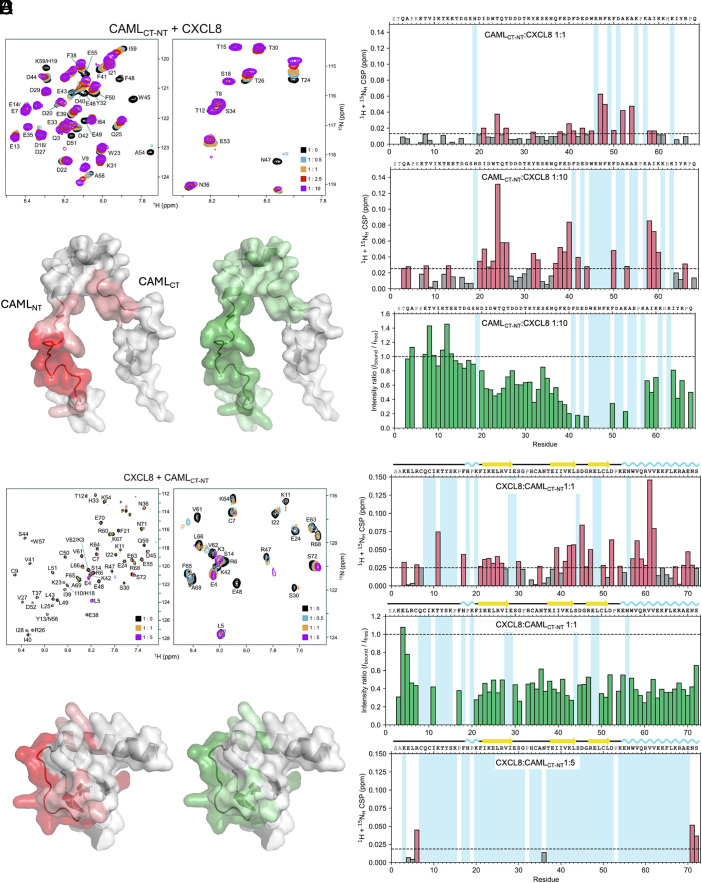
Mapping the cleaved autocatalytic maturation loop (CAML)-CXCL8 binding interfaces. (*A*) Regions of the ^1^H-^15^N HSQC NMR spectra for CAML showing CXCL8 binding-induced chemical shift changes. The unbound peaks are in black, and the final bound peaks are in purple. (*B*) Bar charts of chemical shift perturbations at 1:1 (*Top*) and 1:10 (*Middle*) with the SD indicated as a dotted line. Bar charts of peak intensities at 1:10 are shown at the *Bottom*. (*C*) Surface representation of the modeled CAML structure colored with chemical shift changes (red, *Left*) and intensity loss (green, *Right*). (*D*) Regions of the ^1^H-^15^N HSQC NMR spectra for CXCL8 showing CAML binding-induced chemical shift changes. The unbound peaks are in black, and the final bound peaks are in purple. (*E*) Bar charts of chemical shift perturbations (*Top*) and peak intensities at 1:1 (*Middle*). Total loss of peaks at 1:5 is shown on the *Bottom* in which only N-terminal ELR and C-terminal residues are visible. (*F*) Surface representation of the monomeric CXCL8 structure colored with chemical shift changes (red, *Left*) with CAML as cartoons and intensity loss (green, *Right*).

A construct of the entire disordered SpyCEP_CAML_ region was designed for NMR structural studies. As the CAML_NT_ and CAML_CT_ fragments are close in space at their junctions to the main enzyme, a single 9 kDa polypeptide (with fragment order swapped i.e., CAML_CT-NT_) could be generated with termini that mimic the native CAML of the full-length heterodimeric enzyme (Fig. 2*A*). After completion of NMR assignments for CAML_CT-NT_, a titration with CXCL8 induced significant perturbations to peak positions consistent with the formation of a specific complex. Most chemical shift perturbations (CSPs) were observed in fast-to-intermediate exchange on the NMR timescale, with an observable saturated bound state and a calculated K_d_ of 32.2 ± 3.0 µM. Amide peaks within the acidic, aromatic-rich region of CAML_NT_ (^216^DFDEDWENFEFDAEAEPK^233^) exhibited the largest shifts, highlighting a prominent role (Fig. 3 *A* and *B* and *SI Appendix*, Fig. S7*A*). At high molar ratios, some modest chemical shift changes also occurred within the CAML_CT_ region ^263^HDIDWTQT^270^. Quantification of the intensity reductions also confirmed these regions as interaction sites within CAML_CT-NT_ (Fig. 3 *B* and *C*).

A reverse titration was performed, in which unlabeled 9 kDa CAML_CT-NT_ was introduced into ^15^N/^13^C-labeled CXCL8 and both ^1^H-^15^N/^13^C HSQC NMR spectra were recorded. As with full-length SpyCEP and SpyCEP_NT_ titrations (Fig. 2*B*), the identical global broadening of amide resonances was observed, with virtually all resonances disappearing at saturating levels of the CAML_CT-NT_ (Fig. 3 *D* and *E* and *SI Appendix*, Fig. S7*B*). Residues distal to the CXCL8 dimer interface were the most severely broadened at a lower molar ratio, suggesting that this forms the major CAML_CT-NT_ binding site (Fig. 3 *E* and *F*). The fact that global line broadening was only observed for CXCL8 NMR spectra and not from SpyCEP CAML perspective, which exhibited defined CSPs that saturate, indicates this is not a result of an increase in molecular size (i.e., aggregation). Furthermore, ^1^H DOSY NMR spectroscopy confirmed that no aggregated CXCL8 species are present (*SI Appendix*, Fig. S8*A*). We observed a diffusion constant of 125 μm^2^/s for CXCL8 at 30 μM concentration, consistent with a mixed monomer-dimer population (24). In complex with CAML_CT-NT,_ the measured diffusion constant is similar or faster at 130 to 140 μm^2^/s, despite the severe broadening of NMR linewidths (*SI Appendix*, Fig. S8*A*). Altogether, the NMR data can be best explained by the formation of a “fuzzy” ensemble of CXCL8 conformational states in complex with the SpyCEP CAML, manifesting as multiple distinct chemical shifts being exchange-averaged (25). The faster diffusion constant also suggests that the SpyCEP_CAML_ interaction shifts the CXCL8 dimer equilibrium toward monomer.

We next explored the line-broadening in NMR spectra of side-chains, as they should experience a different motional timescale, and the improved spectral properties would allow chemical shift changes to be followed. While similar global exchange broadening was also observed in ^1^H and ^13^C NMR spectra, chemical shift perturbations (up to Δδ^13^C ~ 0.5 ppm and Δδ^1^H ~ 0.04 ppm) could be followed for ring current shifted groups within the hydrophobic core. (*SI Appendix*, Fig. S8*B*). Although the small downfield ^1^H chemical shift perturbations suggest destabilization of the aromatic and methyl-containing residues within the hydrophobic core, the final state remains close to the native structure and is therefore reminiscent of an unlocked native state (26–28). Unlocked native states, often termed “dry” molten globules (DMGs), are compact intermediates with native-like secondary structure with increased flexibility and weakened side-chain packing, but the core remains dehydrated (26–28). In line with this, no loss in secondary structure in CXCL8 when bound to the disordered SpyCEP CAML_CT-NT_ was detected by CD spectroscopy (*SI Appendix*, Fig. S8 *C* and *D*). Additionally, no increase in ANS fluorescence for the CXCL8–SpyCEP_CAML_ complex (*SI Appendix*, Fig. S8*E*) confirms a compact core that is inaccessible to ANS binding, compared to an ANS-binding positive control for CXCL8 at pH 3 (*SI Appendix*, Fig. S8*F*) (29). Our findings align with recent time-resolved solid-state NMR data on unlocked native states (30), in which chemical shifts revealed a conformation close to the native state, with linewidth analyses indicating increased disorder on the ms to μs timescale.

**Fig. 4.**
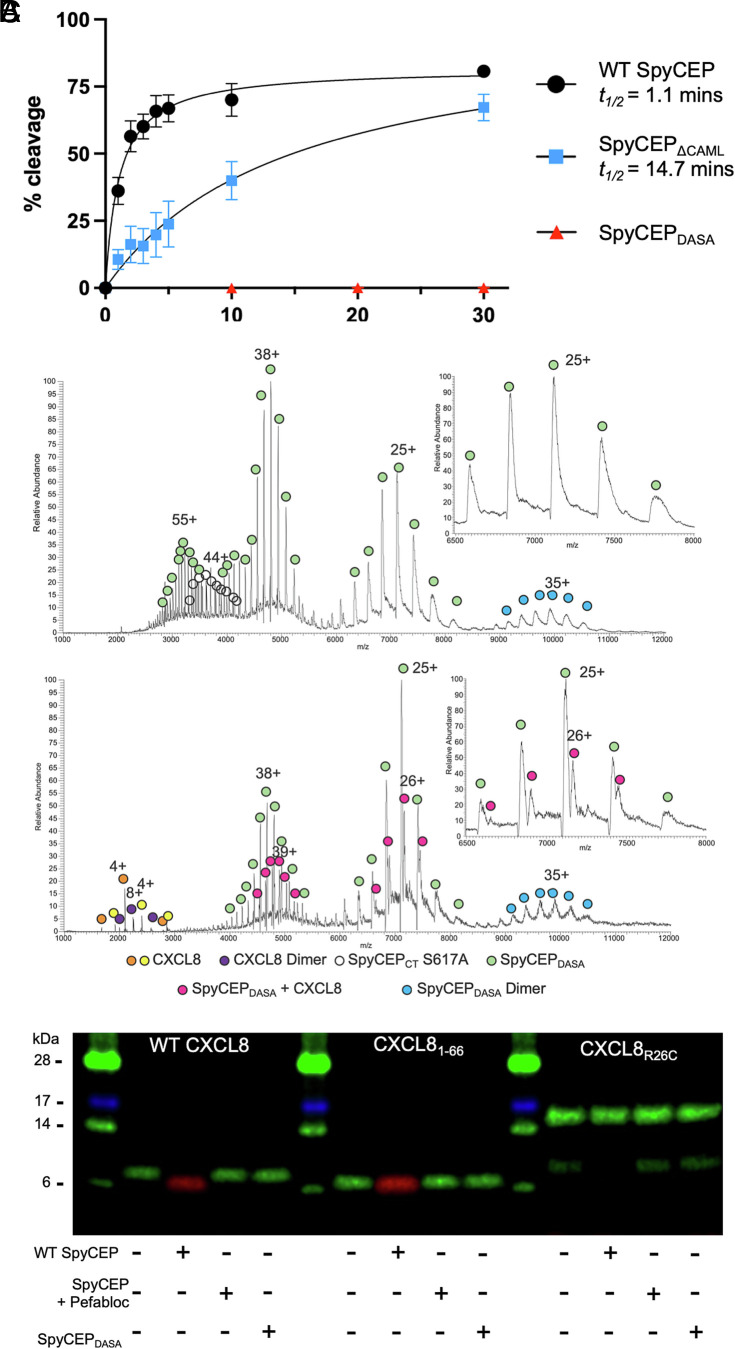
The CAML is required for optimal cleavage and SpyCEP captures monomeric CXCL8. (*A*) Cleavage activity of wild-type SpyCEP, the SpyCEP_ΔCAML_ mutant and the inactive SpyCEP_DASA_ mutant using CXCL8 ELISA. SpyCEP constructs were coincubated with 5 pmol CXCL8 at a 1:10 ratio. Plots show residual CXCL8 over a 30-min room temperature incubation. Reactions were halted at specified timepoints by the addition of Pefabloc to a final concentration of 2 mg/mL. N = 4 experimental replicates, data points show means, error bars represent SE. (*B*) SpyCEP-FL and CXCL8 form a 1:1 complex. Native mass spectrum of full-length SpyCEP (*Top*). Full-length SpyCEP mixed with CXCL8 (*Bottom*). In the *Insets* are zoom-ins of the 6,500 to 8,000 m/Z range. Data presented are representative spectra from two technical repeats. (*C*) CXCL8_WT_, CXCL8_1-66_, and CXCL8_R26C_ were incubated with SpyCEP, SpyCEP WT with 2 mg/mL Pefabloc or SpyCEP_DASA_ mutant at a 2 μM:2 nM (1,000:1) ratio for 16 h at 37 °C. Cleavage products were resolved by SDS-PAGE and revealed by multiplex Western blot (n = 3).

Altogether, we propose that dynamic CAML interactions conflict with the lowest energy conformation of CXCL8 and generate a conformationally frustrated ensemble, which represents an unlocked native-like state. The CXCL8 scissile bond, Q59-R60, lies in an ordered structure near the beginning of the C-terminal helix, and adjacent to a V61-V62 anchor within the hydrophobic core. The CAML-induced opening of the native conformation of CXCL8 would lower the activation energy for attack of the cryptic cleavage site and accelerate catalysis. To test this, we generated an active SpyCEP mutant in which the CAML region was deleted (SpyCEP_ΔCAML_) and performed a comparative cleavage assay with wild-type SpyCEP (Fig. 4*A*). The CXCL8 degradation half-life (*t*_1/2_) increases from 1.5 min for wild-type SpyCEP to 10 min for SpyCEP_ΔCAML_ and confirms an important role for the CAML region, despite no involvement in the catalytic site.

### SpyCEP Interacts with Monomeric and Dimeric CXCL8 But Captures Monomers.

Although our NMR titrations were performed at concentrations where the CXCL8 is predominantly dimeric, we tested whether the SpyCEP CAML could form specific complexes with both CXCL8 monomer and dimers. NMR titrations were repeated using well-characterized “trapped” dimer and monomer mutants; namely the CXCL8_R26C_ disulfide dimer (31) and the monomeric CXCL8_V27P, E29P_ mutant (32). Upon titration into ^15^N-labeled SpyCEP CAML_CT-NT,_ chemical shift perturbations in ^1^H-^15^N HSQC NMR spectra confirm binding to CXCL8 monomers and dimers via the same interaction surfaces (*SI Appendix*, Fig. S9*A*). Although the mutations themselves may alter the measured affinity, the dissociation constants are all in a similar micromolar range (CXCL8-WT K_d_ = 32.2 ± 3.0 µM, CXCL8_R26C_ dimer K_d_ = 75.2 ± 16.8 µM and CXCL8_V27P, E29P_ monomer K_d_ = 107.4 ± 8.9 µM). Furthermore, the reverse titrations show the same CAML-induced global line broadening in CXCL8 variant spectra as in wild-type (*SI Appendix*, Fig. S9*B*).

Protease-associated (PA) domains often play roles in regulating substrate access and specificity of the protease, rather than catalyzing the cleavage of peptide bonds. Notably, the PA domain in the related cell envelope protease from *S. pyogenes*, ScpA (C5a peptidase), specifically recognizes the receptor-binding C-terminus of its substrate, C5a (33). We next assessed whether the PA domain in SpyCEP (residues 424-567 - SpyCEP_PA_) contributes to CXCL8 binding. Although the ^1^H-^15^N HSQC NMR spectrum of ^15^N,^13^C-labeled SpyCEP_PA_ displays all the features of a folded, monomeric PA domain, only ~60% of the expected amides were visible and amenable to assignment, which reflects some inherent conformational dynamics. Nevertheless, we next performed an NMR titration in which the spectrum was monitored in the presence of an increasing amount of wild-type CXCL8 (*SI Appendix*, Fig. S10). Very small CSPs were observed, which were recapitulated but larger in a second titration using the monomeric mutant CXCL8_V27P,E29P_ (*SI Appendix*, Fig. S10). The reverse titration of SpyCEP_PA_ revealed CSPs within the C-terminal helix of CXCL8 (*SI Appendix*, Fig. S10). These data suggest that the PA domain plays a role in the final capture and optimal presentation of a CXCL8 monomer to the protease domain. Together, our interactional data suggest a two-step mechanism for substrate recognition: SpyCEP CAML first fly-casts for CXCL8, which then, together with the SpyCEP PA domain captures the monomer.

To determine the stoichiometry of the final bound state directly, we performed native mass spectrometry (MS) of full-length SpyCEP_DASA_ and its complex with wild-type CXCL8. Charge envelopes for full-length SpyCEP_DASA_ revealed three major species that can be assigned to the C-terminal chain of SpyCEP_DASA_ (151,725 ± 8 Da), together with the full-length SpyCEP_DASA_ heterodimer (177,886 ± 48 Da) and truncated versions without His-tags (Fig. 4*B* and *SI Appendix*, Table S2). In the SpyCEP_DASA_+ CXCL8 complex, dimeric CXCL8 (17,984 ± 1 Da) was observed alongside an additional species (185,955 ± 20 Da), which corresponds to a 1:1 complex. We also observed evidence for a minor tetrameric population of SpyCEP, comprising two heterodimers. No evidence for the CXCL8-bound tetrameric state could be found, and while the functional significance remains unclear, it could represent an autoinhibited state.

Although an initial encounter complex with CXCL8 dimers can be fly-cast by the SpyCEP CAML, the final captured state is monomeric. We therefore predicted that a cleavage reaction involving the covalently locked dimer (31) would not proceed. SpyCEP cleavage reactions were subsequently performed on WT CXCL8, obligate CXCL8_1-66_ monomer, and fixed CXCL8_R26C_ dimer (31). After biochemical confirmation of their oligomeric status, chemokine variants were incubated with either SpyCEP, SpyCEP with the serine protease inhibitor Pefabloc, or SpyCEP_DASA_, then analyzed by SDS-PAGE and also probed by 2-color multiplex Western blot (34), which detects both CXCL8 and the neo-epitope (ENWVQ) following SpyCEP cleavage (Fig. 4*C*) and confirmed by CXCL8 ELISA (*SI Appendix*, Fig. S11), (34). Wild-type CXCL8 and CXCL8_1-66_ monomer were cleaved to completion by SpyCEP within 16 h (Fig. 4*C* and *SI Appendix*, Fig. S11), whereas no cleavage occurred when incubated with SpyCEP_DASA_ or inhibited SpyCEP. In agreement with our prediction, SpyCEP was unable to cleave the locked dimer CXCL8_R26C_.

### Glycan Microarray and NMR Analyses of SpyCEP–GAG Interactions.

As CXCL8 sequesters onto extracellular matrix GAGs in its dimeric form (35) we next asked whether SpyCEP also targets GAGs to facilitate encounter with this major CXCL8 reservoir, possibly via the CAML region. To investigate SpyCEP–GAG binding properties, we first employed glycan microarray experiments using bespoke arrays from the Carbohydrate Microarray Facility at Imperial College London. Specifically, we used a focused GAG oligosaccharide microarray comprising 61 lipid-linked GAG oligosaccharide probes (36), neoglycolipids (NGL) representing diverse classes of GAGs, including hyaluronic acid (HA), chondroitin sulfate (CS) A, B (also known as dermatan sulfate), C, and D, as well as heparin, heparan sulfate (HS), and keratan sulfate (KS). The GAG NGLs probes span a range of oligosaccharide lengths, between 4 and 20 monosaccharide units (*SI Appendix*, Table S3).

**Fig. 5.**
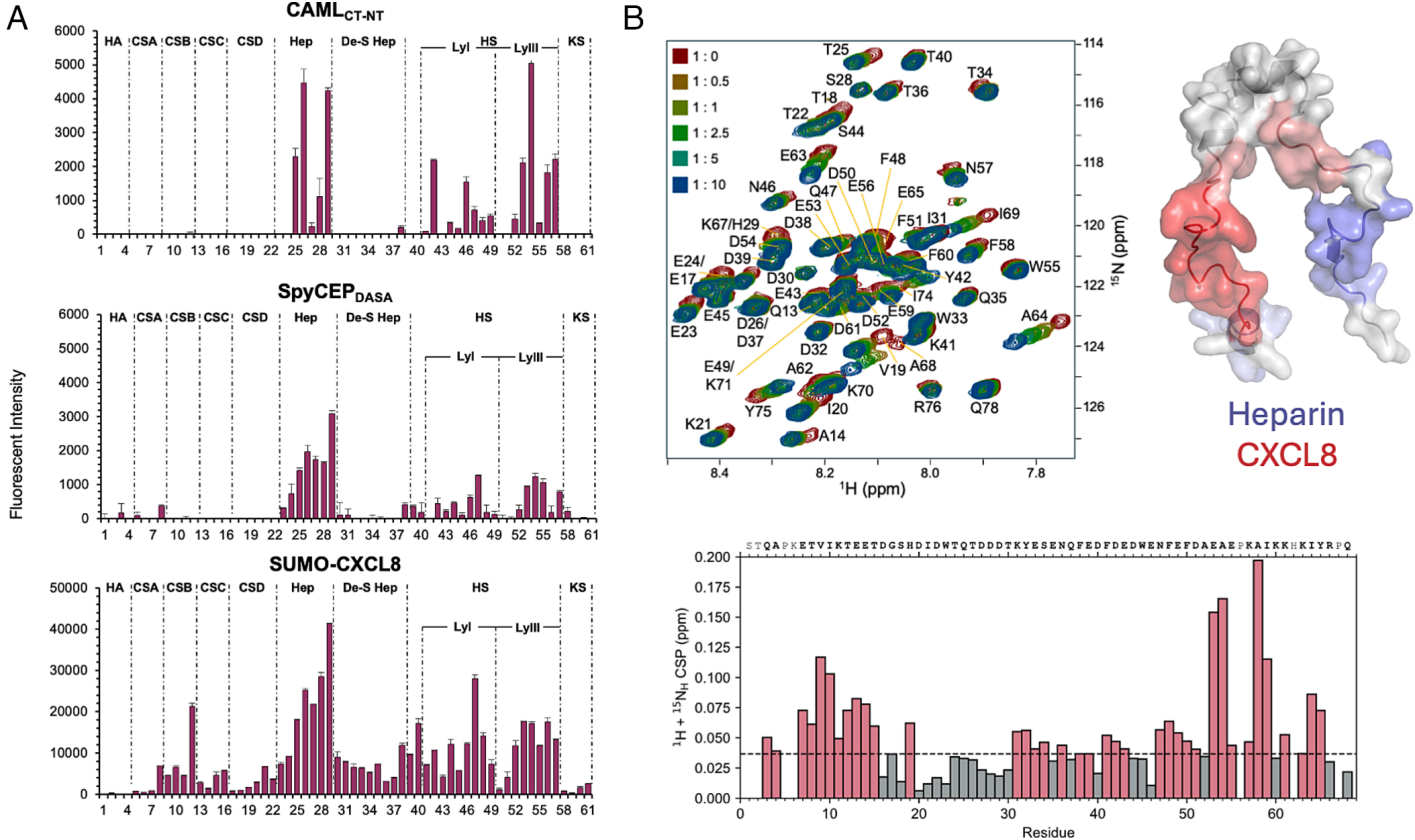
Glycan microarrays and NMR analyses of SpyCEP–GAG interactions. (*A*) Bar chart showing fluorescence intensities of binding of His-tagged SpyCEP CAML_CT-NT_ (*Top*), full-length SpyCEP (*Middle*) and SUMO-CXCL8 (*Bottom*). Error bars represent half of the difference between values for duplicate spots of GAG oligosaccharide probes printed at 5 fmol per spot. (*B*) ^1^H-^15^N HSQC NMR titration of ^15^N labeled SpyCEP CAML_CT-NT_ with unfractionated heparin (*Top Left*), surface representation of CAML colored red for chemical shift changes upon CXCL8 binding, and blue for heparin binding (*Top Right*), and intensity loss bar chart of chemical shift perturbations at 1:10 with the SD indicated as a dotted line (*Bottom*). In the sequence, assigned residues are represented in bold and unassigned with plain text.

Three His-tagged protein samples were chosen, namely SpyCEP_DASA_, CAML_CT-NT_, and CXCL8 in a His–SUMO3 conjugate as a control. SpyCEP_DASA_ bound to heparin oligosaccharides, with signal intensity increasing with oligosaccharide chain length (Fig. 5*A*). SpyCEP_DASA_ also bound probes derived from HS, which are less sulfated than heparin but widely distributed in the extracellular matrix and on cell surfaces. Overall, binding to HS oligosaccharide probes was weaker compared to heparin, which represents the highly sulfated domains of HS. Furthermore, SpyCEP_DASA_ bound less well to site-specific desulfated heparin probes, probes from the CS and KS series, nor to nonsulfated HA probes. This suggests that specific sulfation level and sugar composition are key determinants of SpyCEP binding. Strikingly, the overall GAG-binding profile of SpyCEP_DASA_ closely resembled that of CXCL8, although CXCL8 showed additional binding to CSs, particularly long-chain CSB. These observations indicate that SpyCEP would be targeted to chemokine reservoirs bound to cell-surface GAGs. Notably, CAML_CT-NT_ also bound the same heparin and HS probes confirming that it is a primary GAG-binding domain of the SpyCEP protein.

To map the heparin and HS binding sites at the residues level we next performed ^1^H,^15^N-HSQC NMR titrations with ^15^N-labeled SpyCEP CAML_CT-NT_. Many chemical shift changes (CSPs) occurred upon the addition of unfractionated heparin (average Mw 16.5 kDa), or HS (average Mw 22 kDa), with calculated K_d_ values of 5.94 ± 1.22 µM and 45.10 ± 5.35 µM, respectively. These CSPs mapped to the regions near the termini of CAML_CT-NT_, namely the highly conserved ^250^KETVIKTEET^259^ within CAML_CT_ and ^230^EAEPKAIKKHKIYR^242^ within CAML_NT_ (Figs. 2*C* and 5*B*). Notably, these regions possess all the conserved positive charged residues and a consensus GAG binding motif BBXB in CAML_NT_, where B represents a basic residue (37). The totally conserved ^251^ETVI^254^ motif in CAML_CT_ contributes to heparin binding and not the CXCL8 interaction. As the binding sites for heparin and CXCL8 are largely nonoverlapping across the CAML sequence (*SI Appendix*, Fig. S12*A*), we explored whether the SpyCEP CAML could form a ternary complex. After complexing SpyCEP CAML_CT-NT_ with heparin, CXCL8 was subsequently added (*SI Appendix*, Fig. S12*A*). The peak positions for heparin binding site I in CAML_CT_ were unchanged, suggesting that these regions remain bound to heparin after CXCL8 is added. Peaks in the negatively charged, aromatic-rich region of CAML_NT_ (i.e., ^216^DFDEDWENFEFDAEAEPK^233^) that do not titrate with heparin, showed the expected CXCL8 shifts, indicating complex formation. A few peaks showed an exchange-weighted average of chemical shifts for the heparin and CXCL8-bound complexes, but these lie near the boundaries of their binding sites and likely indicate that CXCL8 and heparin compete for this location (*SI Appendix*, Fig. S12*A*). Many peaks within the heparin site II shifted to new positions in the presence of both heparin and CXCL8, which would be consistent with a ternary complex. Although NMR CSPs do not report directly on interfacial contact in complexes, these data indicate that binding of heparin and CXCL8 to SpyCEP is largely mutually inclusive. We therefore predicted that SpyCEP should maintain its ability to efficiently cleave CXCL8 in the presence of GAGs. Indeed, at heparan sulfate concentrations spanning 0.1 µg/mL to 100 µg/mL, no significant effect on CXCL8 cleavage was observed (*SI Appendix*, Fig. S12*B*).

### Structural Ensemble for the SpyCEP–CXCL8–Heparin Complex.

**Fig. 6.**
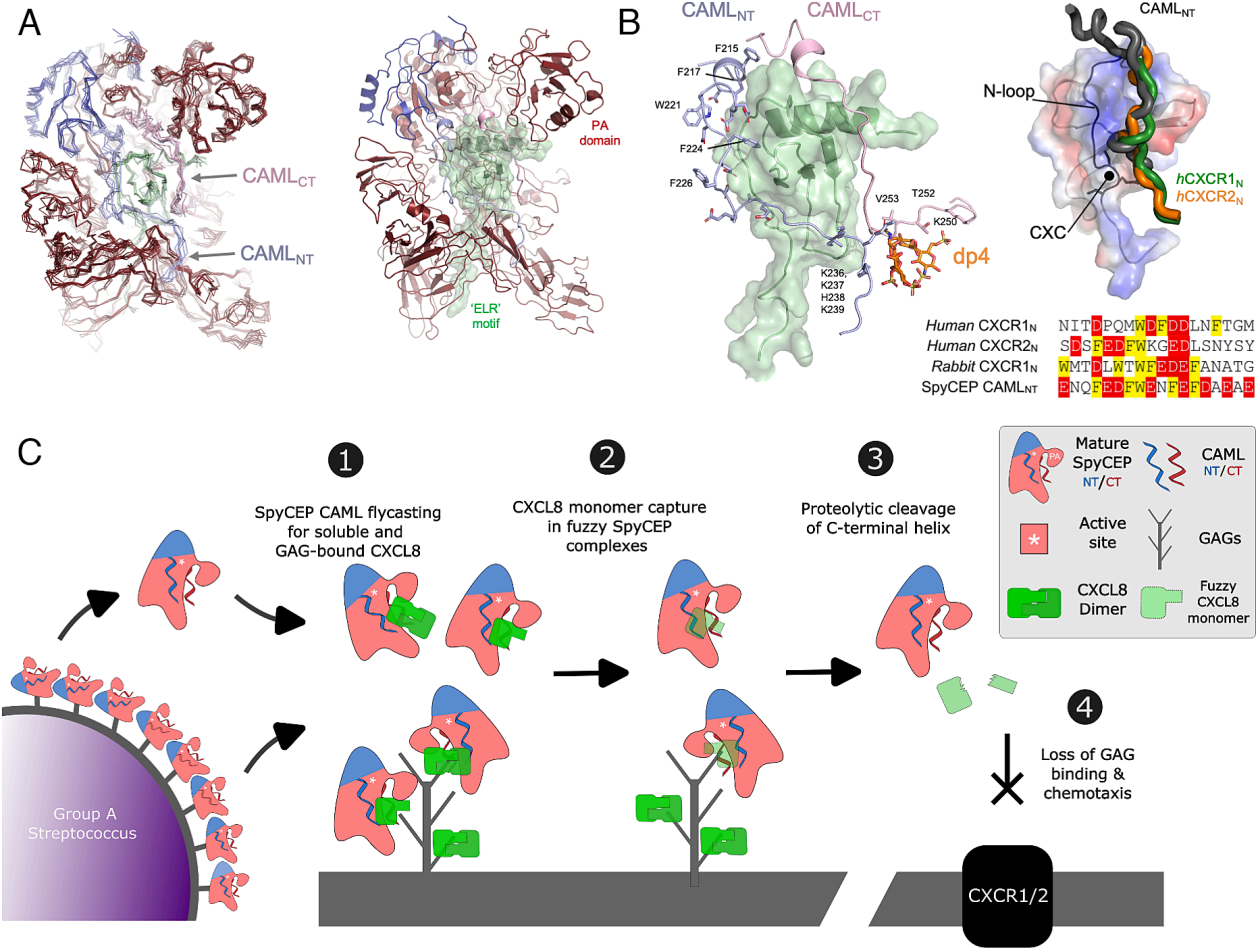
Schematic representation for the mechanism of CXCL8 recognition and degradation by SpyCEP from *S. pyogenes*. (*A*) Ribbon representation of the structural ensemble after refinement of the AF3 model followed by MD simulation with experimental distance restraints (*Left*) with cartoon representation of a representative model (*Right*). (*B*) A zoom of the CAML–CXCL8 interaction surface with heparin dp4 docked using HADDOCK (*Left*) (44) and superposition of the CAML–CXCL8 complex with that of the N-terminal domain from human CXC receptors 1 and 2 together with a sequence alignment including rabbit CXCR1. (*C*) SpyCEP is attached to the bacterial cell and can also be shed into the extracellular milieu. In-solution SpyCEP is shown for clarity. CAML–Cleaved autocatalytic maturation loop, GAGs–glycosaminoglycans, CXCR1/2–CXC motif chemokine receptor 1/2.

An Alphafold (AF)3 (38) model of the full-length SpyCEP heterodimer SpyCEP in complex with monomeric CXCL8 was generated. The involvement of the CAML regions in CXCL8 is predicted by the AF3 mode; however, the precise interactional surfaces are not fully consistent with the NMR mapping. Notably, AF3 invokes the totally conserved ^251^EVTI^254^ motif in CAML_CT_ (Fig. 2), which was determined to be involved in GAG binding by NMR. Furthermore, our NMR titration data also demonstrate that the CXCL8 binding site in CAML_CT_ is shifted nearer to the main body and active site of SpyCEP, which would leave this terminal end free for the confirmed GAG binding. AF3 did predict the preference of SpyCEP for monomeric CXCL8 over dimers (*SI Appendix*, Fig. S13), consistent with our experimental data. To refine the AF3 model, we performed a molecular dynamics simulation guided by distance restraints based on the NMR chemical shift mapping to generate a structural ensemble (Fig. 6*A*) (39). The final model shows CXCL8 located within the large crater delineated by the protease, PA and fibronectin domains and sandwiched between the CAML_NT_ and CAML_CT_ regions. The major interacting region is via the conserved acidic and aromatic region, ^216^DFDEDWENFEFDAEAEPK^233^, within CAML_NT_ (Fig. 6*A*), with the GAG binding regions at the CAML termini free for GAG interaction (Fig. 6*B*). The corresponding binding site on CXCL8 is localized to the CXC motif, N-loop (residues 12 to 18), and third β-strand (residues 44 to 51) and includes both charged and hydrophobic residues. This bears a striking resemblance to the receptor recognition site I where the disordered N terminus of CXC receptors interact (40–42) (Fig. 6*B*). It is worth noting that the CXC motif acts as a conformational switch that mediates coupled dynamic changes throughout the chemokine to tune receptor binding (43). Finally, the CXCL8 cleavage site is positioned proximally to the protease active site with the C-terminal helix extending to the PA domain and the ELR motif extending deep into the void of the SpyCEP cavity.

## Discussion

Bacterial pathogens employ a diverse array of proteases to manipulate host immune defenses, promoting infection and persistence (45). Among these, proteases that selectively target chemokines are particularly important, as they disrupt leukocyte recruitment and suppress inflammatory responses. *S. pyogenes* (Group A *Streptococcus*) strains have evolved a conserved virulence factor, SpyCEP, which possesses the unique ability to inactivate a specific group of CXC chemokines, most notably CXCL8, by removing the GAG-binding region within the conserved C-terminal helix (4–7). While the dynamics of the C-terminal helix of CXCL8 show local unwinding of the last 6 residues (K67–S72) in the monomer (32, 46), which could assist cleavage, the scissile bond (Q59-R60) lies upstream of this within an ordered helical structure. Furthermore, the C-terminal helix is stabilized and dynamically restricted within the dimeric and GAG-bound reservoirs, the latter of which enables the establishment of stable concentration gradients for neutrophil chemotaxis (32, 35, 46).

Our working model for SpyCEP function invokes a multistep mechanism, which combines the manipulation of substrate conformational dynamics, monomer sequestration, and localization to chemokine reservoirs. SpyCEP first fly-casts for initial encounters with its substrate, in monomer or dimer form, using its cleaved autocatalytic maturation loop (CAML), which is intrinsically disordered and affords a large capture radius. Second, the acidic, aromatic-rich region of the disordered CAML targets an allosteric hotspot on the chemokine to form a frustrated conformationally heterogeneous (fuzzy) complex, resembling an unlocked native state, that is coupled to dimer dissociation. The substrate binding pocket delineated by the protease (PR), protease-associated (PA), and C-terminal domains (rIg1/2) (19) can accommodate a CXCL8 monomer for capture and cleavage. In vitro, CXCL8 cleavage has been observed for a CAML deletion mutant SpyCEP (47), but work presented herein confirms that it is significantly slower than wild-type (Fig. 4*A*). Strikingly, the related protease CspA in Group B *Streptococcus*, which lacks the CAML region and possesses a divergent PA domain, is unable to cleave CXCL8 (48).

At infection sites, CXCL8 can accumulate to micromolar levels, whereas circulating CXCL8 in systemic infection is generally in the 1-10 nM range (35, 49). Within infected tissue, CXCL8 would be largely sequestered as dimers and monomers on matrix glycosaminoglycans (GAGs), which have a high effective concentration, with a small free monomer and dimer pool (13). To counter this, invasive *S. pyogenes* strains (particularly those with CovR/S mutations) decorate their surfaces with a unique “brush” of tethered SpyCEP proteases (50) that would reach an effective pericellular concentration of up to 100 µM, as well as being actively released into the extracellular milieu. Estimating the steady-state flux of SpyCEP under different scenarios reveals that its ability to i) promote CXCL8 dimer dissociation and ii) function efficiently within GAGs accelerates substrate depletion by an estimated 5- to 30-fold near inflamed infection sites (*SI Appendix*, Table S6). This would generate a local “black hole” of chemokine clearance around each bacterium and a neutrophil-free zone, which matches the characteristic paucity of neutrophils observed in invasive *S. pyogenes* infections (7). Released SpyCEP eventually diffuses through the GAG network, and its high catalytic efficiency (*K_M_* = 82 nM and *k_cat_* = 1.6 s^−1^) means that it is well adapted to low CXCL8 concentrations in the circulation and at distant tissues where it acts as a broader chemokine sink. Substrate dimerization and GAG binding would have less influence here, and SpyCEP can function via the monomer-only pathway to efficiently flatten CXCL8 gradients according to first-order enzyme kinetics (*SI Appendix*, Table S6) and assist in the systemic spread of infection (7, 15, 51). Accordingly, wild-type infections show sharply reduced serum chemokine levels compared with SpyCEP-deficient strains.

Structural plasticity is a general property of CXC chemokines as it plays important roles in modulating dimerization and receptor activation (13, 32, 40–42, 46, 52–54). The CXC motif and the N-loop provide the predominant interaction surface with the receptor N terminus, and this region acts as a conformational on-switch that modulates dynamics throughout the chemokine to refine receptor binding (43). While dynamically driven fine-tuning allows diversity in the functional signals, it also creates a vulnerability that could be exploited. The SpyCEP CAML hijacks CXCL8’s coupled dynamic network by engaging with the same hotspot on CXCL8 (Fig. 6*B*), and trapping CXCL8 monomers in a heterogeneous conformational state for cleavage and removal of the C-terminal helix (Fig. 6*C*).

Interestingly, the modulation of dynamics was observed in human CXCL8 in complex with either rabbit and human CXCR1 N-domains, and this induced dimer dissociation (18, 55). Binding of CXCL8 to the human CXCR1 N-domain promotes formation of an ordered complex on the ps-ns timescale, but with conformational changes coupled to increased ms-μs dynamics in other parts of the molecule (46, 56, 57). Indeed, dimer dissociation is important for CXCR1 binding, as monomeric CXCL8 is the high-affinity ligand (58, 59). A striking sequence feature of human and rabbit CXCR1 interface is an aromatic and acidic rich region within the first half of the receptor N-domain (Fig. 6*B*). The CXCL8-interaction region within the SpyCEP CAML is a mimic of this and is further elaborated with aromatic and acid residues (Fig. 6*B*). In our working hypothesis, this expansion allows for transient, alternative contacts with the allosteric hotspot of CXCL8, switching on coupled dynamics throughout the molecule. SpyCEP can cleave several members of the ELR^+^CXC chemokines family, which all act on CXCR1/2 receptors. A consensus cleavage site has been proposed (16), and its location within the same region of the C-terminal helix suggests a similar mechanism of chemokine remodeling would be necessary (*SI Appendix*, Fig. S14*A*). Predictions of the complexes between SpyCEP and other ELR^+^CXC chemokine reveal the same interface with an ensemble of conformations for the CAML (*SI Appendix*, Fig. S14*B*). The fuzzy nature of the SpyCEP CAML–chemokine interaction would facilitate SpyCEP adaptation to the different ELR^+^CXC substrates (8, 16).

The SpyCEP CAML also provides potential to localize the enzyme to cell-surface GAGs with a binding specificity mirroring that of its chemokine substrates. Studies using a murine model of soft tissue infection showed that expression of SpyCEP by *S. pyogenes* (or even when expressed in nonpathogenic *Lactococcus lactis*) led to a significant increase in bacteria in the regional lymph nodes, liver, spleen, and bloodstream, compared to strains without SpyCEP (7). This demonstrates that the protease facilitates the spread of bacteria from a local site of infection into the wider body. SpyCEP exists in both cell-wall anchored and soluble forms (6, 60), therefore it can not only cleave soluble CXCL8 in the vicinity of the bacterium but will also concentrate at specific chemokine reservoirs throughout the local tissue, independent of the main bacterial adherence points (Fig. 6*C*). CXCL8 inactivation over a wider radius around the infection site than diffusion alone might allow, would more effectively dismantle the transendothelial chemokine gradient needed to recruit neutrophils from the bloodstream into the infected tissue. This would facilitate dissemination from the localized infection site to deeper tissues and other organs, which is characteristic of severe invasive diseases like necrotizing fasciitis or bacteremia.

Our work showing that SpyCEP exploits a natural vulnerability in chemokine dynamics illustrates how intrinsic disorder and host glycan exploitation converge to overcome structural barriers—a strategy distinct from other proteases. More broadly, it suggests that other pathogen proteases may employ similar strategies to process “protected” host targets, opening avenues for anti-infective drug design. Furthermore, the crucial role of CAML in CXCL8 inactivation, its conservation across SpyCEP orthologs and a vaccination study showing protection for a minimal peptide epitope from the CAML (9) suggest it could be exploited in the design of a smarter multiantigen GAS vaccine.

## Materials and Methods

### Production of Full-Length SpyCEP, CAML, PA Domain, and Human CXCL8.

SpyCEP N-(A34-Q244) and C-terminal (S245-A1613) domains were cloned into pET-28b (Novagen) utilizing N- and C-terminal hexa-histidine tags, respectively. These constructs encompass the *emm*1 *S. pyogenes* SpyCEP ectodomain, excluding the leader peptide and LPXTG motif. Two inactive mutants (D151A and S617A) were produced with the Q5 Site-Directed Mutagenesis Kit (New England Biolabs) and verified by DNA sequencing. Recombinant protein was produced by growing transformed *Escherichia coli* BL21 (DE3) cells (C2527, New England Biolabs) at 37 °C until an optical density at 600 nm of 0.7 was reached. Protein expression was induced by addition of 100 mg/L (0.42 mM) isopropyl β-d-thiogalactopyranoside (IPTG) and the cultures were grown at 18 °C overnight and purified using methods described previously (19). The methodology to produce recombinant human CXCL8 and its mutants was adapted from methods described (61), and later improved using SMT3 fusion for higher efficiency cleavage with ULP1, which produced untagged CXCL8. Both CXCL8 V27P/E29P (32) and CXCL8_1-66_ monomer mutants (58, 59) were used in this study. These mutants disrupt the dimer interface and trap CXCL8 in a monomeric state and are interchangeably in in vitro biochemical and cellular assays with equivalent activity. For NMR spectroscopy, we used the V27P/E29P full-length monomer mutant as it contains the full sequence length. The R26C dimer (31) which locks CXCL8 dimer with a covalent disulfide bond was used in both NMR and functional assays.

### Cryoelectron Microscopy (EM).

The SpyCEP_DASA_+3F2G10 (334.7 kDa) complex was prepared by mixing SpyCEP at 5 µM with 6 µM mAb (1:1.2 ratio) and incubated on ice for 30 min. The antibody complex was isolated using gel filtration on a Superdex S200 10/300 column equilibrated in 20 mM Tris pH 8, 200 mM NaCl, and validated by 4 to 20% SDS-PAGE stained in Coomassie blue. SpyCEP_DASA_–CXCL8 complexes were crosslinked with 0.4-2% formaldehyde and the experimental design was based on (23, 62). Crosslinked SpyCEP_DASA_+CXCL8 (~184.7 kDa) was purified in the same buffer, but SpyCEP_DASA_+CXCL8 (184.7 kDa) used 40 mM tris pH 7.5, 150 mM NaCl. Cryogrids were prepared using the Vitrobot Mark IV system in accordance with *SI Appendix*, Table S3.

Screening was performed using a Tecnai T12 TWIN microscope equipped with a TVIPPS 4 K CCD camera operated at 120 kV. Parameters included −3 µm defocus, pixel size of 2.234 Å/pixel (52,000×), and 25 electrons/Å^2^ dose for 1 s exposure. Multigrid screening and movie collection utilized a Glacios-TEM equipped with a Falcon IV 4 K Direct Electron Detector operated at 200 kV. Movie collection was performed using defocus values from −1 µm to −2.5 µM, a pixel size of 1.5 Å/pixel (79,000×) or 1.16 Å/pixel (100,000×), and 40 to 50 electrons/Å^2^ for 10 to 20 s exposure. Collection with a Titan Krios-TEM operated at 300 kV used a Gatan K3 Direct Electron Detector, defocus values from −1 µm to −2.5 µM, a pixel size of 0.67 Å/pixel (120,000×), and 40 electrons/Å*2* for 10 to 20 s exposure.

Datasets underwent patch motion correction, patch CTF estimation, and curation for micrographs with CTF fit resolutions better than 4.5 Å, defocus values between 1.0 to 2.5 μm, and minimal motion drift. Particle picking and selection generally followed a three-step process: 1) blob picking and 2D classification to generate initial templates, 2) template-based picking and 2D classification to refine templates, and, if possible, 3) Topaz training for deep-learning-based particle picking and 2D classification. Following particle selection, ab-initio models were generated and improved using nonuniform refinement. The resulting electron density maps were used to fit a molecular scaffold, if the resolution was sufficient. This workflow served to improve the angular diversity of 2D classes and is summarized for each map in *SI Appendix*, Figs. S2–S5.

### NMR Spectroscopy.

All spectra were recorded on a Bruker Avance III HD 800 MHz or Bruker 600 MHz standard bore NMR spectrometer equipped with a triple-resonance cryoprobe. Spectral assignment of [*U*-^15^N, ^13^C]- labeled SpyCEP CAML_CT-NT_ was performed at 623 µM in 20 mM Na phosphate, 50 mM NaCl, pH 7.0, 10% D_2_O at 298 K. CBCA(CO)NH and HNCACB (63) triple resonance spectra, alongside hNCAnH were acquired for backbone assignment. The assignment covers 60 of the 64 residues, excluding the hexa-histidine tag and proline residues. Spectral assignment of [*U*-^15^N, ^13^C]- labeled SpyCEP PA domain was performed in a similar manner. Spectral assignment of CXCL8 was obtained from two sources (61, 64). [ILV-^1^H_3_-^13^C, ^2^H]-labeled SpyCEP_DASA_ and SpyCEP_CT_ S617A were also produced (α-ketobutyric acid, CDLM-7317; α-ketoisovaleric acid, CDLM-4611.) Titrations with (CAML_CT-NT_/CXCL8, SpyCEP_PA_/CXCL8, SpyCEP_PA_/CXCL8_V27P/E29P_, CAML_CT-NT_/GAG) were performed with 30 to 170 µM [*U*-^15^N, ^13^C]- labeled protein in 20 mM Na phosphate, 50 mM NaCl, pH 7.0, 10% D_2_O at 298 K. Titrations with (CXCL8/SpyCEP_CT_ S617A, CXCL8/SpyCEP_NT_ D151A, CXCL8/SpyCEP_DASA_) were performed with 153 to 170 µM [*U*-^15^N, ^13^C]- labeled protein in PBS 7.4 pH, 10% D_2_O at 298 K. Titrations with (SpyCEP_DASA_/CXCL8) were performed with 30 µM [ILV-^1^H_3_-^13^C, ^2^H]-labeled protein in PBS 7.4 pH, 100% D_2_O at 298 K. NMR spectra were recorded to molar ratios of 1:10 or until saturation. The GAGs utilized in NMR studies were unfractionated heparin sodium salt (3 to 30 kDa; PHR8927, Merck) and heparan sulfate (9 to 35 kDa; GAG-HS01, iduron). Combined chemical shift perturbations of the individual amide pairs were calculated using Δδ = [Δδ^2^_H_ + (Δδ_N_/R_scale_)^2^]^1/2^, with an R_scale_ value of 5.0 (65). All spectra were processed with NMRPipe (66) and analysis was performed using CCPN Analysis version 2.4 (67) and version 3.3.2.1 (68). Graphical summaries were produced with in-house Python scripts (version 3.11.9) using the matplotlib library.

### Native Mass Spectrometry.

Protein mixtures were buffer exchanged to 500 mM Ammonium Acetate (Sigma-Aldrich) on Bio-Spin P-6 gel columns (Bio-rad). Typically, 2 to 3 µL of protein sample was loaded onto a borosilicate glass capillary (Thermo Scientific, ES380). Proteins were injected into a Thermo Scientific Q-Exactive UHMR Quadropole-orbitrap hybrid instrument equipped with Direct Mass Technology (Thermo Scientific) with an attached nano-electrospray ionization (ESI) source. Source and HCD gas used was 99.999% pure nitrogen. The following instrument conditions were used: AGC target of 1e6, spray voltage of 1.2 kV, maximum injection time of 100 ms, number of microscans at 1, resolution of 12,500, noise threshold of 3, trapping gas pressure of 8 - UHV pressure of 3.06e^−10^ mbar, in-source collision-induced dissociation (CID) at 50 V, capillary temperature at 250 °C. The instrument was calibrated with 2 mg/mL CsI solution. Data acquisition and analysis was performed on the Thermo Scientific Tune and Xcalibur software packages.

### Glycan Microarray Analysis.

Glycan Microarray analyses were performed using the NGL-based microarray platform (69) on a focused GAG oligosaccharide array containing 61 lipid-linked GAG oligosaccharide probes. Details of the glycan probe library, microarray fabrication, glycan-binding protein samples are provided in *SI Appendix*, Table S5 in accordance with the Minimum Information Required for A Glycomics Experiment (MIRAGE) guidelines for the reporting of glycan microarray data (70). Slides were blocked for 1 h with the blocking solution (10 mM HEPES, pH 7.4, 150 mM NaCl containing 1% w/v BSA [Sigma7030], 0.02% w/v blocker Casein [Pierce], and 5 mM CaCl_2_). Protein samples (SpyCEP full-length and SpyCEP CAML_CT-NT_, at 150 µg/mL; SUMO-CXCL8 at 50 µg/mL) were then overlaid onto the arrays for 90 min. Detection was carried out using a mouse monoclonal anti-poly-histidine antibody (Sigma SAB4200620) followed by a biotinylated anti-mouse IgG (Sigma B7264), both at 10 µg/mL for 60 min. The final detection step involved a 30-min incubation with streptavidin-Alexa Fluor 647 (Molecular Probes) at a concentration of 1 µg/mL. Imaging and data analysis are described in the Supplementary MIRAGE document (*SI Appendix*, Table S5).

### Assays for the Detection of CXCL8 Cleavage by SpyCEP.

These assays have recently been described in detail (71). Briefly, chemokines and SpyCEP were incubated at 37 °C in a final volume of 20 µL PBS (pH 7.4) using a 1:1,000 enzyme:substrate ratio. Control digests lacked SpyCEP, contained SpyCEP preincubated with Pefabloc (2 mg/mL), or the catalytically inactive SpyCEP_DASA_. Reactions were terminated by heating in DTT-containing sample buffer and following resolution on SDS-PAGE assayed by multiplex Western blot. Intact CXCL8 was detected by an anti-CXCL8 mAb (MAB208, R&D Systems) and cleaved CXCL8 by an anti-ENWVQ rabbit antisera generated in house. Imaging was with an Odyssey XF imager. Western blot data were verified by use of a CXCL8 sandwich ELISA (R&D Systems, Abingdon, UK).

## Supplementary Material

Appendix 01 (PDF)

## Data Availability

Study data are included in the article and/or *SI Appendix*. The coordinates of the atomic model of SpyCEP from Streptococcus pyogenes complexed with an anti-PA domain monoclonal antibody 3F2G10 have been deposited in the Protein Data Bank with accession code 31MR (72). The cryo-EM maps of the SpyCEP complexes with anti-PA domain monoclonal antibody 3F2G10 and the anti-N-terminal monoclonal antibody 10B6C10 have been deposited with accession codes EMD-58555 (73), and EMD-58529 (74).
