# Supplementary material for: SpyCEP dismantles neutrophil immunity via disorder-driven chemokine remodeling and GAG targeting
Source: Proc Natl Acad Sci U S A. 2026 Jul 9;123(28):e2520164123. doi: 10.1073/pnas.2520164123 (PMC13367824; doi:10.1073/pnas.2520164123)
Supplement: Supplementary file 1 — Appendix 01 (PDF) [file pnas.2520164123.sapp.pdf]

Supporting information

SpyCEP dismantles neutrophil immunity via disorder-driven chemokine remodeling and GAG targeting

Rikin J. Lau[1,2], Sean P. Giblin[3], Andra Sugar[1], Antonio Di Maio[4], Giulio Tassini[5], Kristin Huse[2,6], Dror Chorev[7], Yuan Chen[4], Grace Ho-Yan Wu[1], Camilla Berg Huemer[1,2], Seung Yon Kim[1], Jayden Matthews[1], Bel Muloud[1], Lu Chen[1], Sophie McKenna[1,2], Yingqi Xu[1], Luisa Massai[5], Chiara Muzzi[5], Xhenti Ferhati[5], Francesca Necchi[5], Danilo Gomes Moriel[5], Ten Feizi[4], Yan Liu[4], James E. Pease[3], Shiranee Sriskandan[2,6], Steve Matthews[1,2#]

[1]Department of Life Sciences, Imperial College London, South Kensington Campus, SW7 2AZ. UK
[2]Centre for Bacterial Resistance Biology, Imperial College London, London SW7 2AZ, UK
[3]National Heart and Lung Institute, Imperial College London, London W12 0NN, UK
[4]Institute of Reproductive and Developmental Biology, Department of Metabolism, Digestion and Reproduction, Faculty of Medicine, Imperial College London, London W12 0NN, UK
[5]GSK Vaccines Institute for Global Health, Via Fiorentina 1, 53100 Siena, Italy
[6]Department of Infectious Disease, Imperial College London, London W12 0NN, UK
[7]C.T.M. Technologies and Materials Ltd, Ness-Ziona, 3 Pinkhas Sapir Street, 7403626 Israel.

[#]Correspondence: s.j.matthews@imperial.ac.uk

This PDF file includes:

Supporting text
Figures S1 to S14
Tables S1 to S6
SI References

Supporting Information Text

Materials and Methods

Purification of full length SpyCEP, CAML and PA domain

The constructs were purified by resuspending cellular pellets (20 mM Tris pH 8, 200 mM NaCl, 20 mM imidazole) supplemented with a cOmplete, EDTA-free protease inhibitor tablet (Roche). The cells were disrupted by sonication and the lysate was clarified by centrifugation at 38,000g, 4 °C for 30 min. The clarified lysate was loaded onto a 5 ml HisTrap FF crude column (GE Healthcare) equilibrated with buffer A and eluted with an equivalent buffer containing 500 mM imidazole. Final separation was achieved with a Superdex 75 or 200 10/300 (increase) in accordance with MW (GE Healthcare) equilibrated with 20 mM Na phosphate pH 7.0, 50 mM NaCl. SpyCEP $CAML_{CT\text{-}NT}$ (S245-S278—E211-Q244) and PA (Q424-K567) domains were cloned into pET-28b and produced as above.

Production of recombinant human CXCL8

In-house generated CXCL8WT (residues $CXCL8_{1\text{-}66}$ and $CXCL8_{R26C}$ were expressed solubly using SUMO fusion technology in E. coli SHuffle T7 Express (C3029, New England Biolabs) and purified by IMAC and SEC after SUMO excision with ULP1. The methodology to produce recombinant human CXCL8 was adapted from methods described (40), and later improved using SMT3 fusion for higher efficiency cleavage with ULP1, which produced untagged CXCL8 . The product was verified as greater than >95% pure by SDS-PAGE, and biological activity was confirmed by neutrophil chemotaxis assays. The activity of the R26C CXCL8 variant was verified in binding assays and chemotaxis assays and found to be comparably active to WT CXCL8 (Fig. S10-12).

NMR Spectroscopy

Diffusion ordered spectroscopy (DOSY) was performed by monitoring the proton signal of unlabelled CXCL8/CAML at 1:0, 1:0.5, and 1:1 ratios, with CXCL8 at constant concentration of 100 µM. We used a gradient ramp from 1% up to 91% in 18 linear steps. Experiments were performed in 50 mM NaCl, 20 mM NaP, 100% D_2O. Standard two-dimensional (2D) ^{1}H-^{13}C HSQC and HMQC spectra were recorded for titrations of ^{13}C-labelled CXCL8 with $SpyCEP_{CAML}$ and ^{13}C-methyl isoleucine full-length SpyCEP with CXCL8, respectively.

Production of anti-SpyCEP monoclonal antibodies

Anti SpyCEP monoclonal antibodies 3F2G10 and 10B6C10 are produced as murine IgG1 by Takis s.r.l. using murine hybridomas produced after the immunization of CD1 mice with SpyCEP antigen. mAbs are purified using a protein G purification protocol by Takis s.r.l. then quantified by UV. The quality of the batches is attested by HPLC and SDS-PAGE. Finally, antibodies are sterile filtered 0.22 µm in PBS solution (pH 7.4) and, without additives (e.g. no sodium azide), were stored at -70°C.

Cross-linking of the SpyCEP-CXCL8 complex

$SpyCEP_{DASA}$-CXCL8 complexes were crosslinked with formaldehyde, targeting lysine/arginine residues, and validated by SDS-PAGE. A range of 0.4-2% formaldehyde was assessed. To produce the crosslinked sample utilized in cryo-EM studies, $SpyCEP_{DASA}$ was incubated with CXCL8 at 2% formaldehyde for 3 hours in PBS 7.4 pH, quenched with 0.5 M ammonium bicarbonate for 10 min, and purified using SEC. The covalent complex was separated by boiling at 100 degrees for 5 minutes prior to SDS-PAGE (4-20%), which confirmed the presence of CXCL8.

Cell culture and transfection

The mouse pre-B cell line Ba/F3 was purchased from the DSMZ-German Collection of Microorganisms and Cell Cultures and passaged in RPMI supplemented with 10% FBS and 10 ng/ml mouse IL-3. pCDNA3 plasmids encoding HA-tagged variants of human CXCR1 and CXCR2 were purchased from the cDNA Resource Center (www.cdna.org) and introduced into the Ba/F3 cells as previously described (1). 48 hrs following transfection, cultures were selected in fresh media containing in 1mg/mL G418 and grown to confluency, after which clones were isolated by limiting dilution, expanded and assessed for expression by flow cytometry using a PE-conjugated anti-HA mAb (Clone 16B12, BioLegend, UK).

Competition binding assays

This was a modification of an assay previously described by Schoofs and colleagues (2). Ba/F3 hCXCR1 and hCXCR2 transfectants were resuspended in assay buffer (PBS, 0.1% BSA, 0.05% NaN_3) at a concentration of $5x10^6$ cells/mL. CXCL8-AF647 was purchased from Almac (Craigavon, UK). Binding assays were carried out on a polypropylene 96 well plate in duplicate and consisted of 50µL of cells in a final volume of 200µL with a 2.5nM final concentration of CXCL8-AF647 in the presence or absence of increasing concentrations of unlabelled chemokine, diluted in assay buffer. After a 30-minute incubation at RT in the dark, plate was centrifuged at 400g to pellet cells and pellets washed with 400uL of assay buffer. After another round of centrifugation, cell pellets were resuspended in 200 µL of 1% paraformaldehyde dissolved in PBS and transferred to FACS tubes. Analysis was carried out on a FACS Calibur (BD Biosciences, UK). Data are presented as the percentage of control binding which is calculated as the fluorescent intensity observed in the absence of competing ligand, following subtraction of baseline fluorescence.

Modified Boyden chamber chemotaxis assays

Prior to use, Ba/F3 transfectants were supplemented with Sodium Butyrate (Sigma-Aldrich, UK) at a final concentration of 10mM. Overnight culture in the presence of sodium butyrate enhances the transient expression of chemokine receptors in this system (1). Dilutions of CXCL8 variants were made in assay buffer (RPMI + 0.1% BSA) and plated in the wells of a ChemoTX chamber with a 5 µm pore size (Neuroprobe Inc., Gaithersburg, MD). Migration was allowed to proceed for 5hr at 37C. 5% CO2 after which cells migrating into the lower well were enumerated with Cell Titer Glo (Promega, UK). Data are expressed as a percentage of the input cells.

Realtime TAXIScan chemotaxis assays

Human neutrophils were isolated from whole blood obtained from a subcollection of the Imperial College Tissue Bank, taken from informed, consenting healthy normal subjects. Neutrophils were freshly isolated by negative selection using the MACSxpress neutrophil isolation kits according to the manufacturer's instructions, followed by up to three RBC lysis steps using hypo/hypertonic saline solutions. For the real time analysis of migrating neutrophils, a 12-channel TAXIScan was employed and used according to the manufacturer's protocol (Effector Cell Institute, Tokyo. Japan). One µl of a suspension containing $5x10^5$ neutrophils/ml was loaded into each chamber and following alignment of the cells at one end of the terrace, 1 µl of CXCL8 was added to the opposing end of the terrace (260 µm away) and cells were allowed to migrate along the ensuing chemokine gradient for 1hr at room temperature. Sequential image data were captured every minute as individual jpegs which were subsequently processed with ImageJ (National Institutes of Health), equipped with the manual tracking (Fabrice Cordelieres, Institut Curie, Orsay (France) and chemotaxis tool plugin (3).

Individual experiments consisted of triplicate conditions for each chemokine and data illustrated are collated from an equal number of experiments as highlighted in the figure legend. For each neutrophil, the velocity and directionality of migration were calculated via the chemotaxis tool plugin. Directionality is defined as the ratio of Euclidian distance:accumulated distance travelled. A value of 1 represents migration in a perfectly straight line.

Statistical Analysis

Statistical analyses were carried out using Prism 6 (GraphPad Software, La Jolla, CA) and the tests are noted in the figure legends. * = P<0.05, **= P<0.01, ***= P<0.001 and ****= P<0.0001.

Molecular dynamics simulations and heparin docking

Molecular dynamics (MD) simulations were performed with CABSflex 3.0 server using the flexible setting and experimental intermolecular distance restraints (4). Heparin dp4 was docked on to a representative model using the HADDOCK approach and ambiguous distance restraints between NMR mapped residues and dp4 atom (5).

Circular Dichroism

Circular Dichroism (CD) measurements were performed to monitor changes in the secondary structure of CXCL8 upon interaction with $CAML_{CT-NT}$. A titration series was prepared by mixing 40 µM of CXCL8 with $CAML_{CT-NT}$ at molar ratios from 1:0 to 1:5, in 10 mM Na phosphate pH 7, to a final volume of 200 µL. Each sample was loaded into a quartz cuvette, and CD spectra were recorded using a Chirascan V100 (Applied Photophysics) in the far-UV range of 210-260 nm. Data

below 210 nm were excluded due to excessive noise. Spectra were baseline-corrected by omitting CXCL8 from the reaction mixtures while retaining $CAML_{CT\text{-}NT}$, accounting for background CD contributions from the titrant peptide.

ANS fluorimetry

Fluorescence measurements were performed to monitor changes in the protein core hydrophobicity of CXCL8 upon interaction with $CAML_{CT\text{-}NT}$. A stock solution of 8-anilino-1-naphthalenesulfonic acid (ANS) was dissolved in DMSO at 20 mg/mL (~67 mM). A working concentration of 100 µM ANS was used in all fluorescence assays to minimise DMSO content in the samples. A titration series was prepared by mixing 20 µM CXCL8 with $CAML_{CT\text{-}NT}$ at molar ratio from 1:0 to 1:5. All samples were prepared in 10 mM Na phosphate pH 7, with 4 µL of ANS stock, to a final volume of 200 µL. Fluorescence intensity was measured using a Varioskan LUX multimode plate reader (Thermo Scientific). Each sample was loaded onto a 96-well black-walled, clear-bottom plate (Thermo Scientific). ANS was excited at 375 nm and emission spectra were recorded from 400 to 600 nm. Spectra were baseline-corrected by omitting CXCL8 from the reaction mixtures and retaining $CAML_{CT\text{-}NT}$ and ANS, accounting for background fluorescence from the titrant peptide. Control experiments also included CXCL8 in 10 mM Na phosphate pH 3, 10 mM Na phosphate pH 7 with 4 M urea, and 10 mM Na phosphate pH 7 with 8 M urea.

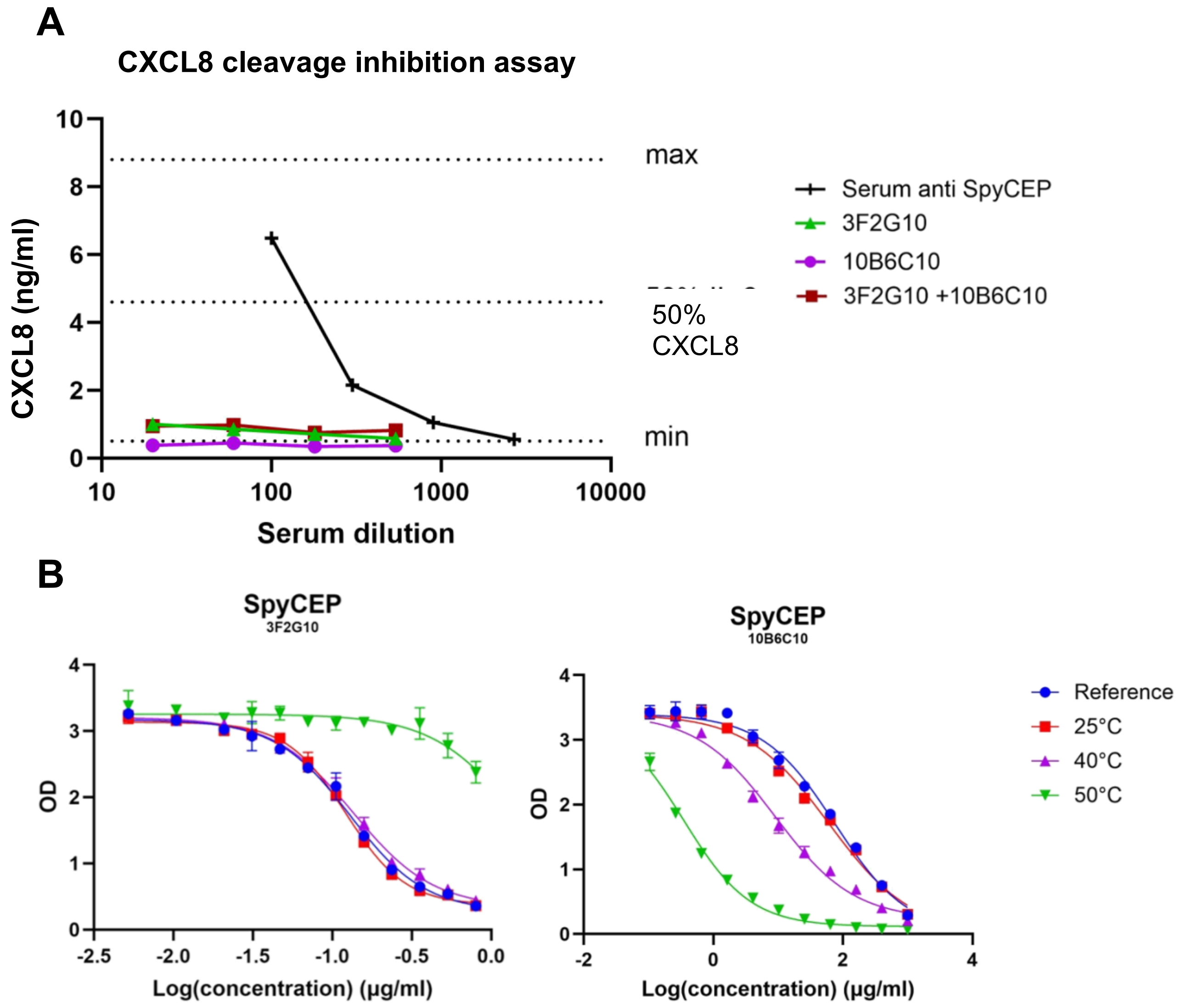

Fig. S1 mAb characterisation A) IL8 cleavage inhibition assay. The functionality of anti-SpyCEP mAbs is attested by an IL8 cleavage inhibition assay, as here indicated. Four consecutive three-fold step dilutions are assayed starting from 1:5 dilution for mAbs and from 1:25 dilution for standard serum, using a Dulbecco's phosphate-buffered saline (DPBS) with 5% bovine serum albumin (BSA) in 25 µl of volume. Then 25 µl of wild type recombinant SpyCEP at the concentration of 20 ng/ml are added to each well (final concentration of 5 ng/ml) and plates are incubated at 4°C for 5 min. Then 50 µl of human IL-8 at the concentration of 20 ng/ml (final concentration of 10 ng/ml) are added and plates incubated for two hours at 37°C. The final dilution of each sample in the first well is 1:20 for mAbs and 1:100 for standard serum once all components are added to the plate. The integrity of IL-8 is then quantified by a human IL-8 Immunoassay ELISA kit (Invitrogen). B) Competitive ELISA (cELISA) assay of anti-SpyCEP mAbs. Antibody binding to $SpyCEP_{DASA}$ after thermal stress (25°C, 40°C, and 50°C) for 5 / 7 days is evaluated by cELISA assay for both 3F2G10 and 10B6C10 mAbs. Binding curves could underline loss (shift to the right) or gain (shift to the left) of the mAb affinity to the antigen after different stress temperatures, compared to the reference condition.

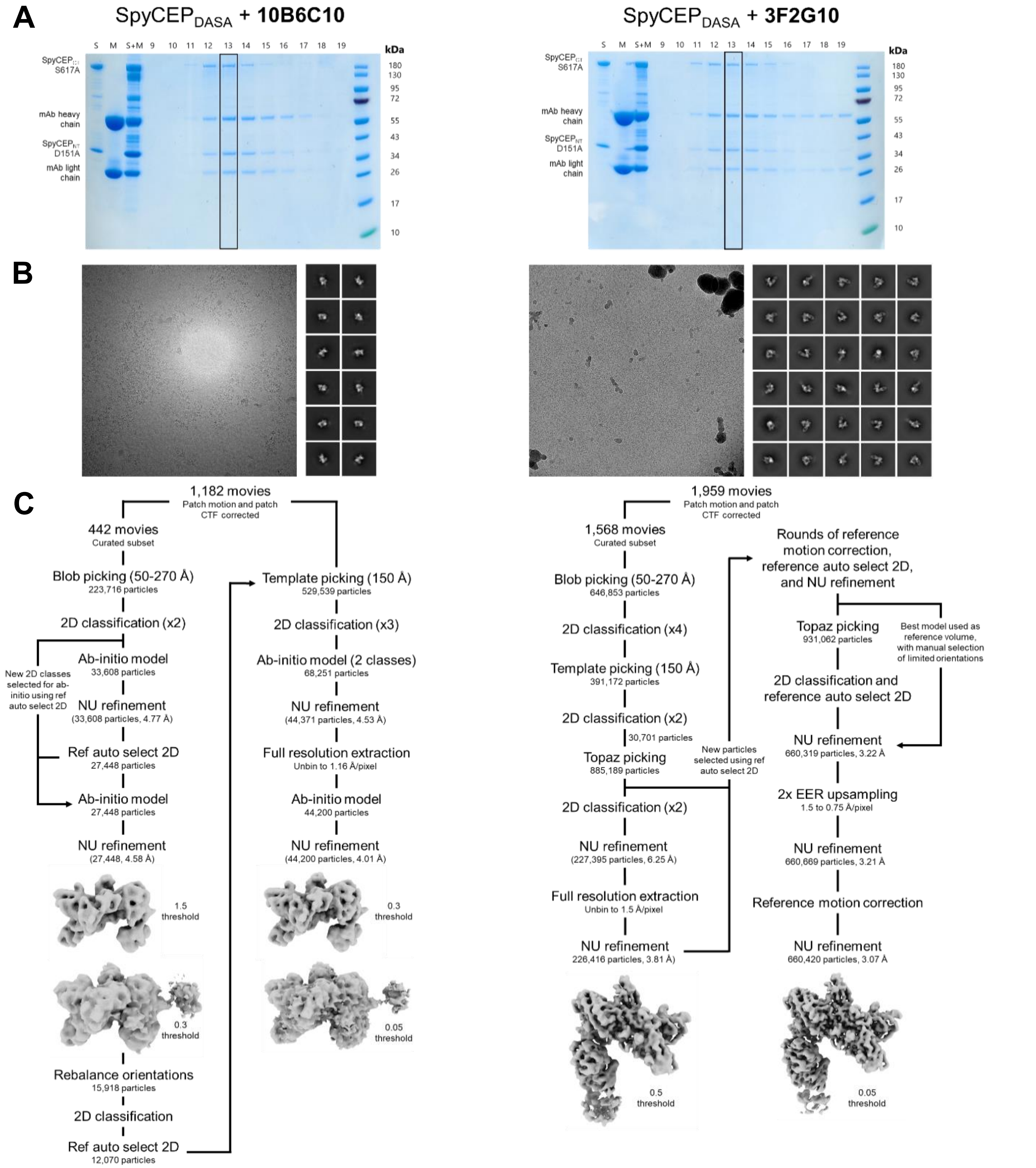

Fig. S2 CryoEM pipeline for SpyCEP-antibody complexes (A) Representative Coomassie-stained SDS-PAGE of purified protein complexes: $SpyCEP_{DASA}$+ 10B6C10 and $SpyCEP_{DASA}$+ 3F2G10. The box represents the fraction used for cryo-EM. (B) Representative micrograph and 2D classes for either dataset. (C) Cryo-EM processing pipeline, showing key 3D maps.

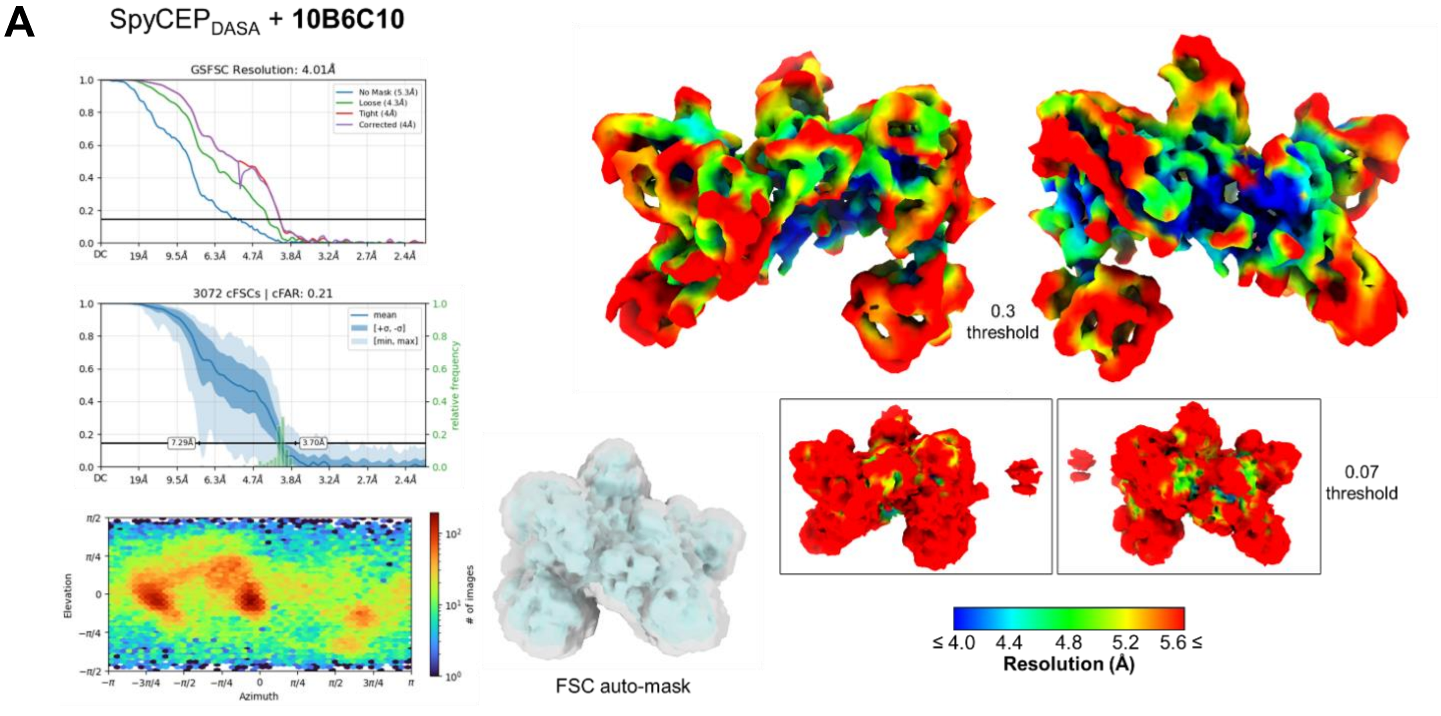

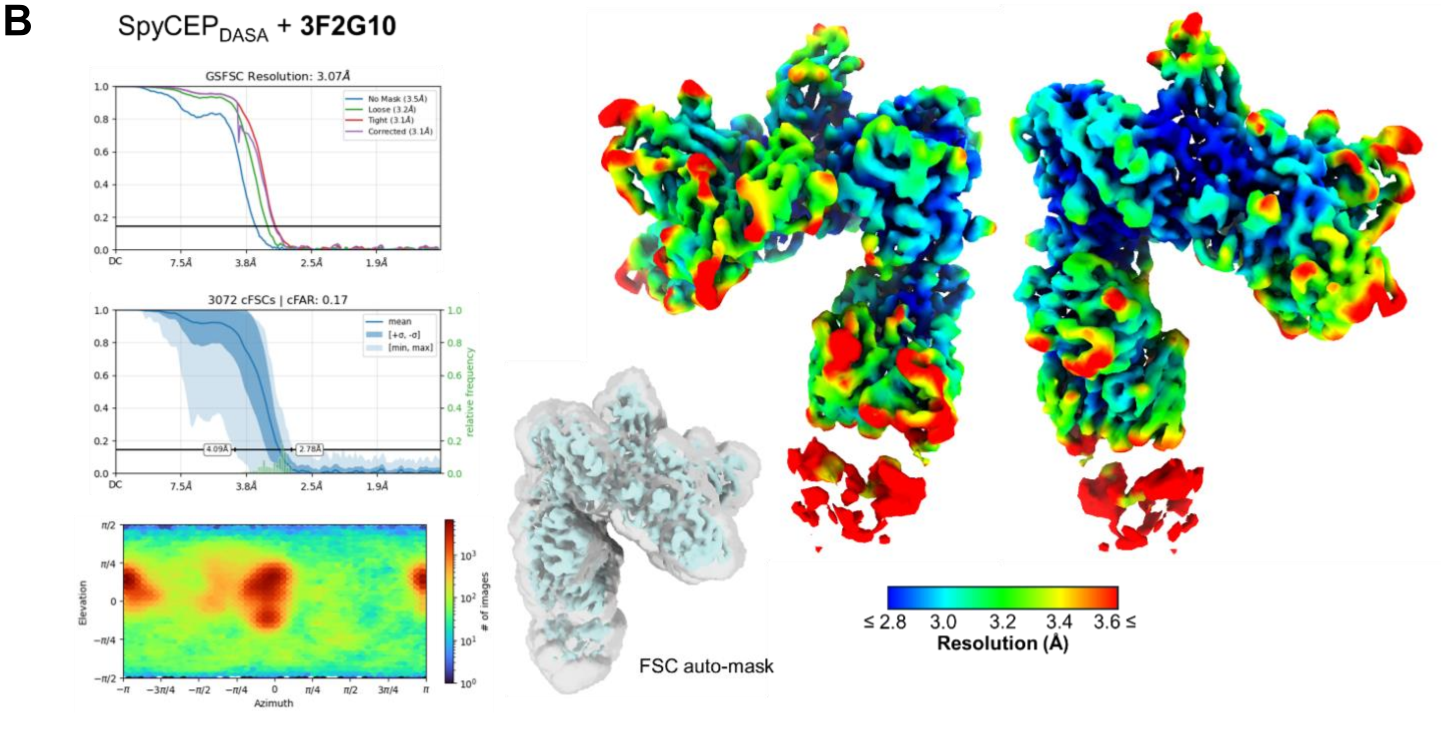

Fig. S3 CryoEM resolution analysis for SpyCEP-antibody complexes Fourier shell correlation (FSC) plots between half-maps, directional (3D FSC) resolution estimate for the final map, orientation distribution plot, mask used to determine the average resolution at FSC 0.143, and local resolution estimates for the final map. (A), SpyCEP$_{DASA}$+ 10B6C10; (B), SpyCEP$_{DASA}$+ 3F2G10.

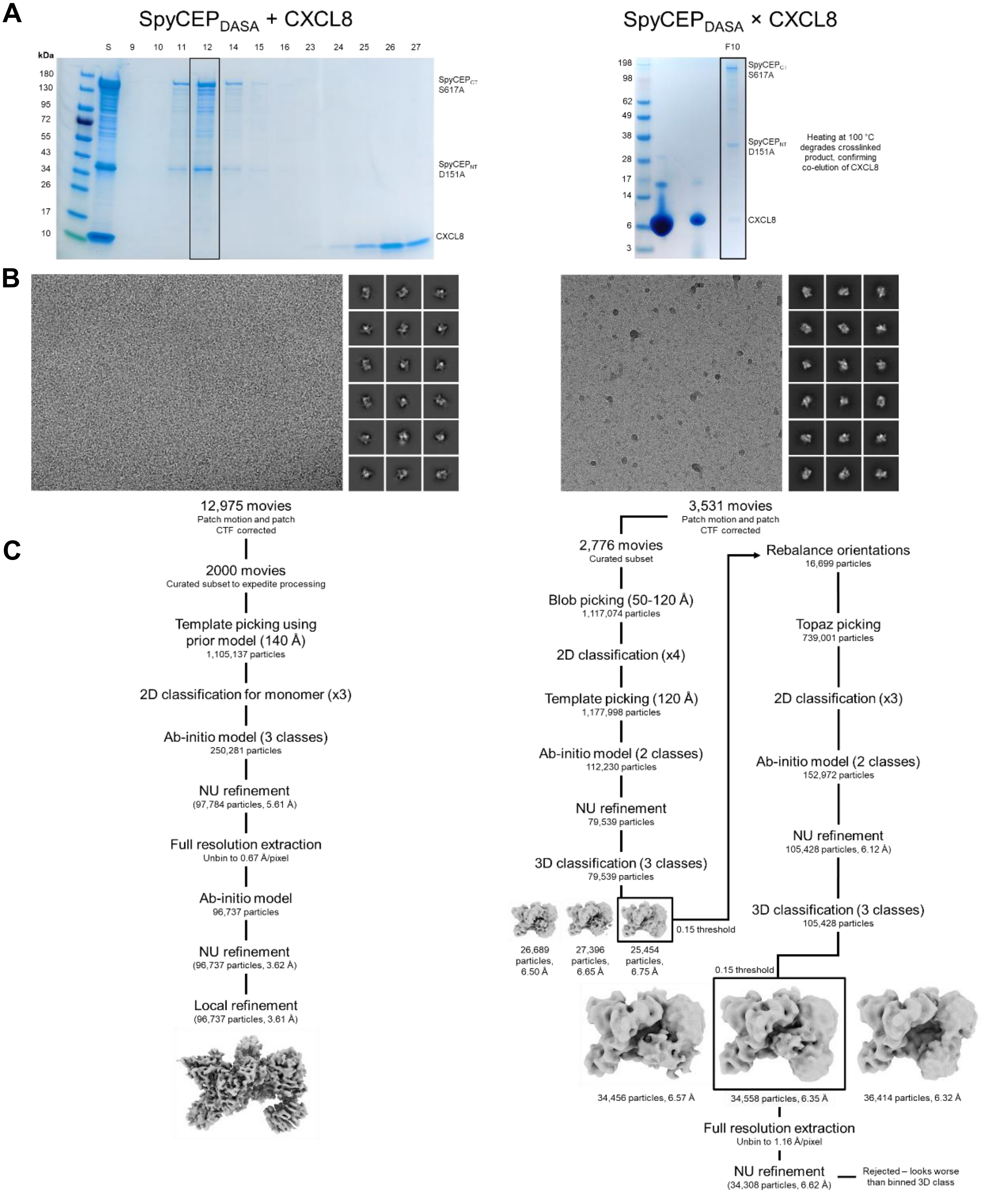

Figure S4 CryoEM pipeline for the SpyCEP-CXCL8 complexes (A) Representative Coomassie-stained SDS-PAGE of purified protein complexes: SpyCEP$_{DASA}$+ CXCL8 (left) and covalently cross-linked SpyCEP$_{DASA}$ × CXCL8 (right). The box represents the fraction used for cryo-EM. (B) Representative micrograph and 2D classes for either dataset. (C) Cryo-EM processing pipeline, showing key 3D maps.

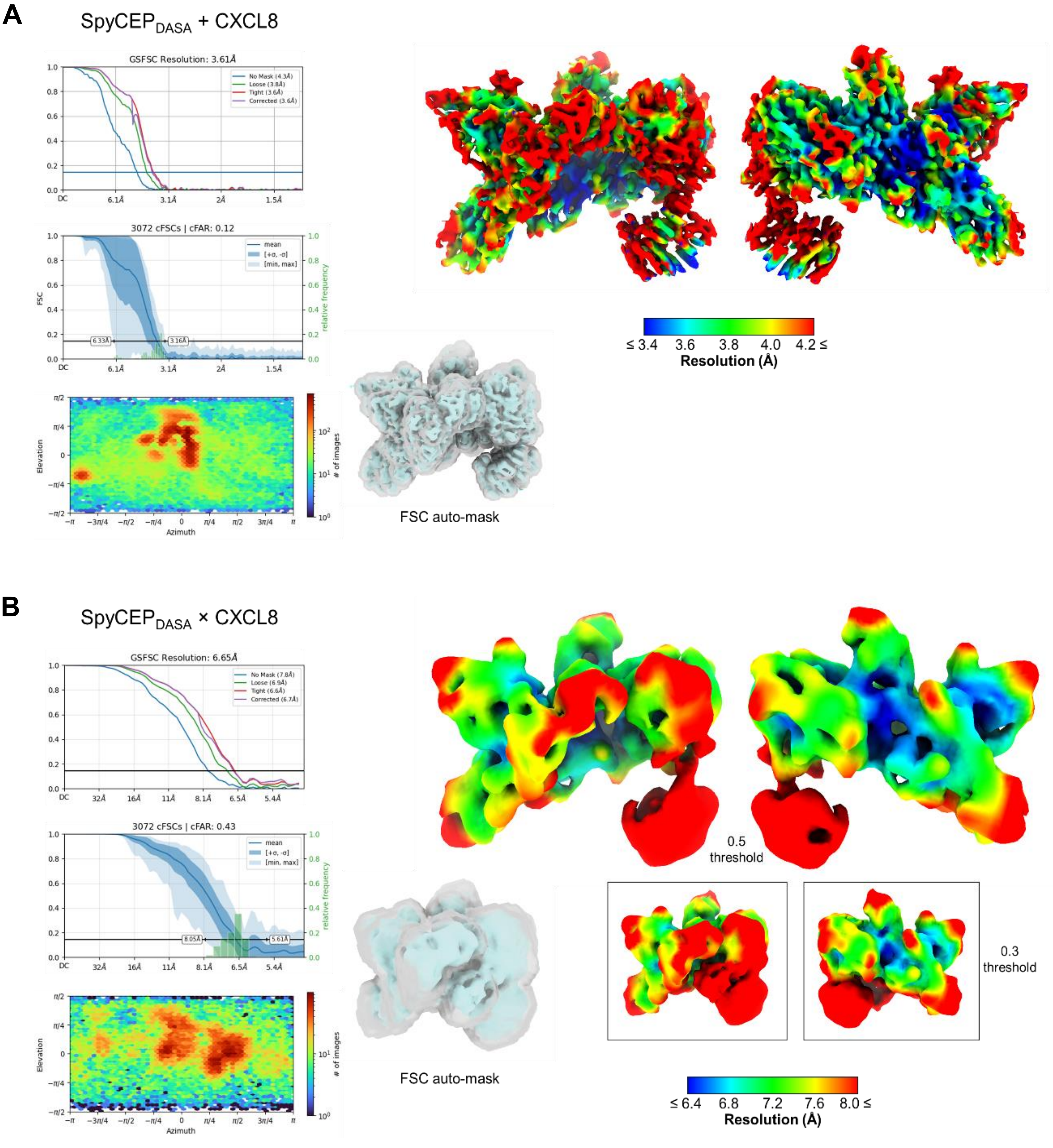

Fig. S5 CryoEM resolution analysis for SpyCEP-CXCL8 complexes Fourier shell correlation (FSC) plots between half-maps, directional (3D FSC) resolution estimate for the final map, orientation distribution plot, mask used to determine the average resolution at FSC 0.143, and local resolution estimates for the final map. (A), $SpyCEP_{DASA}$+ CXCL8; (B), covalently cross-linked $SpyCEP_{DASA}$ × CXCL8.

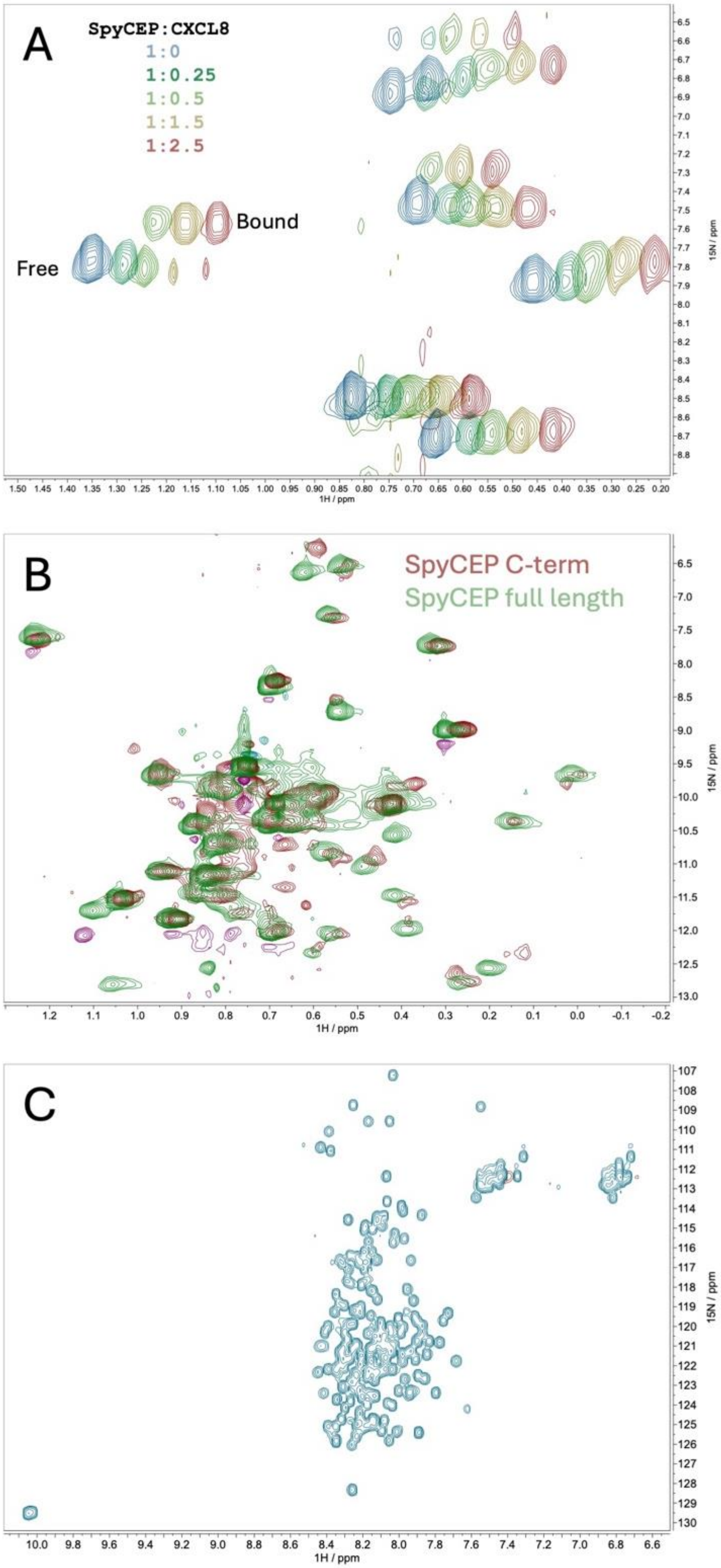

Fig. S6 NMR spectra of SpyCEP N- and C-terminal fragments. A). Shifted overlay of the Isoleucine region from the ^{1}H-^{13}C methyl TROSY of ^{13}C-methyl isoleucine labelled full-length SpyCEP$_{DASA}$ in the presence of increasing amounts of CXCL8 (K_d ~500 nM) B) ^{1}H-^{13}C methyl TROSY spectrum of isoleucine methyls for the SpyCEP$_{CT}$ S617A = alone (mauve) and as the heterodimeric complex with SpyCEP$_{NT}$ D151A (green), illustrating it retains a similar structure in the absence of the N-terminal fragment. C) ^{1}H-^{15}N HSQC of SpyCEP$_{NT}$ D151A alone, illustrating its intrinsic disorder in the absence of the C-terminal fragment.

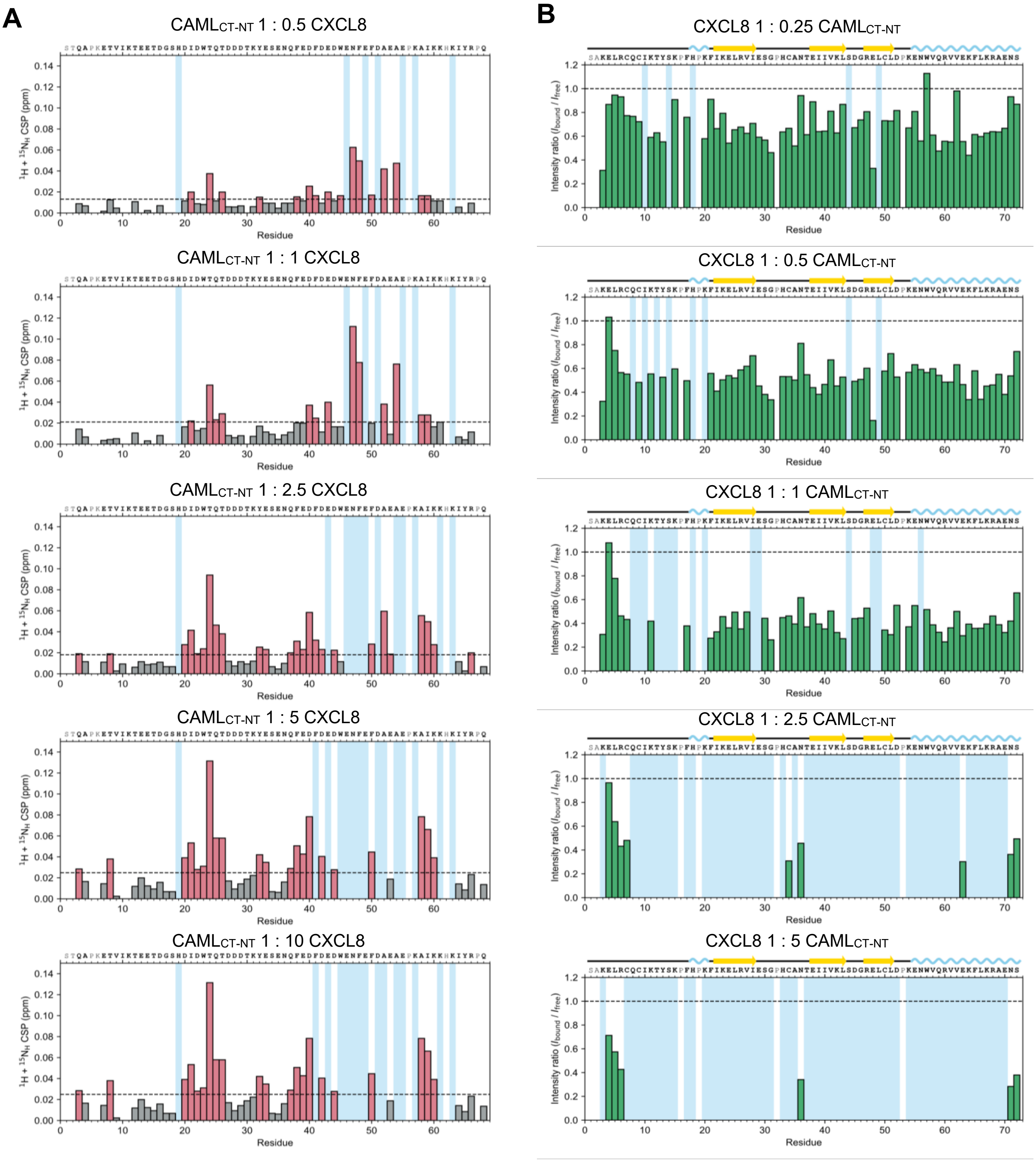

Fig. S7 Mapping the CAML$_{CT-NT}$-CXCL8 binding interfaces A) Bar charts of chemical shift perturbations for ^{1}H-^{15}N HSQC spectra of SpyCEP CAML$_{CT-NT}$ in the presence of increasing amounts of CXCL8 with the standard deviation indicated as a dotted line. B) Bar charts of peak intensities for ^{1}H-^{15}N HSQC spectra of CXCL8 in the presence of increasing amounts of SpyCEP CAML$_{CT-NT}$ with no change indicated as a dotted line. Blue shaded bars represent peaks broadened beyond detection at that titration point.

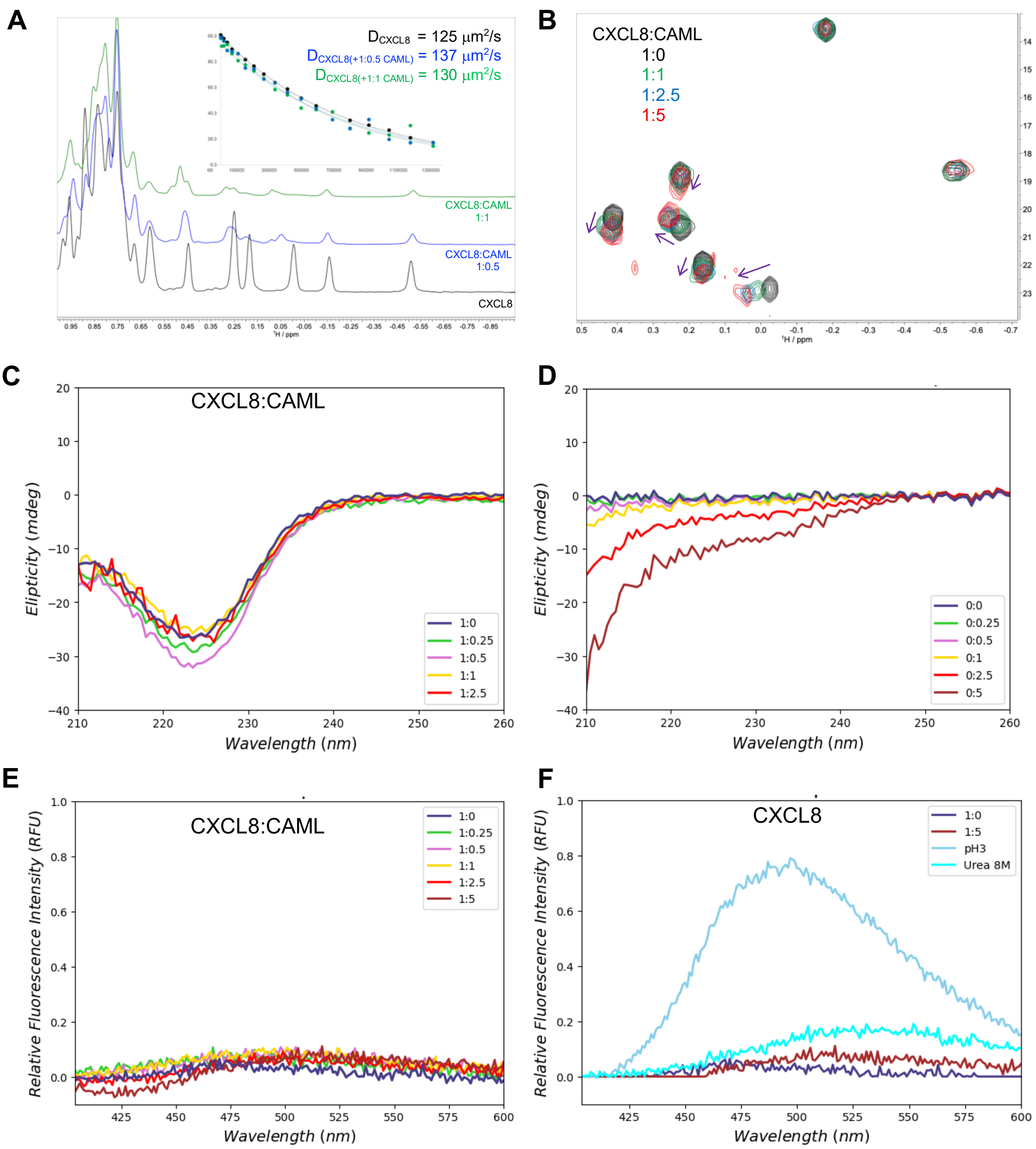

Fig. S8 Fluorescence spectroscopy of CAML$_{CT-NT}$-CXCL8 interaction A) ^{1}H DOSY NMR measurement of the CXCL8 diffusion constant in the presence of CAML$_{CT-NT}$. B) Methyl group region of ^{1}H-^{13}C HSQC spectra of CXCL8 with increasing CAML$_{CT-NT}$ showing shifts and line broadening. C) CD titration spectra of CXCL8 with CAML$_{CT-NT}$. D) CD spectra of free CAML$_{CT-NT}$ at various concentrations, confirming its disordered state. E) Fluorescence spectra (400-600 nm) recorded after excitation at λ = 375 nm for the titration of CXCL8 with CAML$_{CT-NT}$ in the presence of 100 µM ANS. F) Comparison of fluorescence spectra for titration points 1:0 and 1:5 (from E) with that of a positive ANS-binding CXCL8 control (represented by CXCL8 at pH 3, which remains appreciably folded (6)) and a fully denatured negative control in urea.

A

B

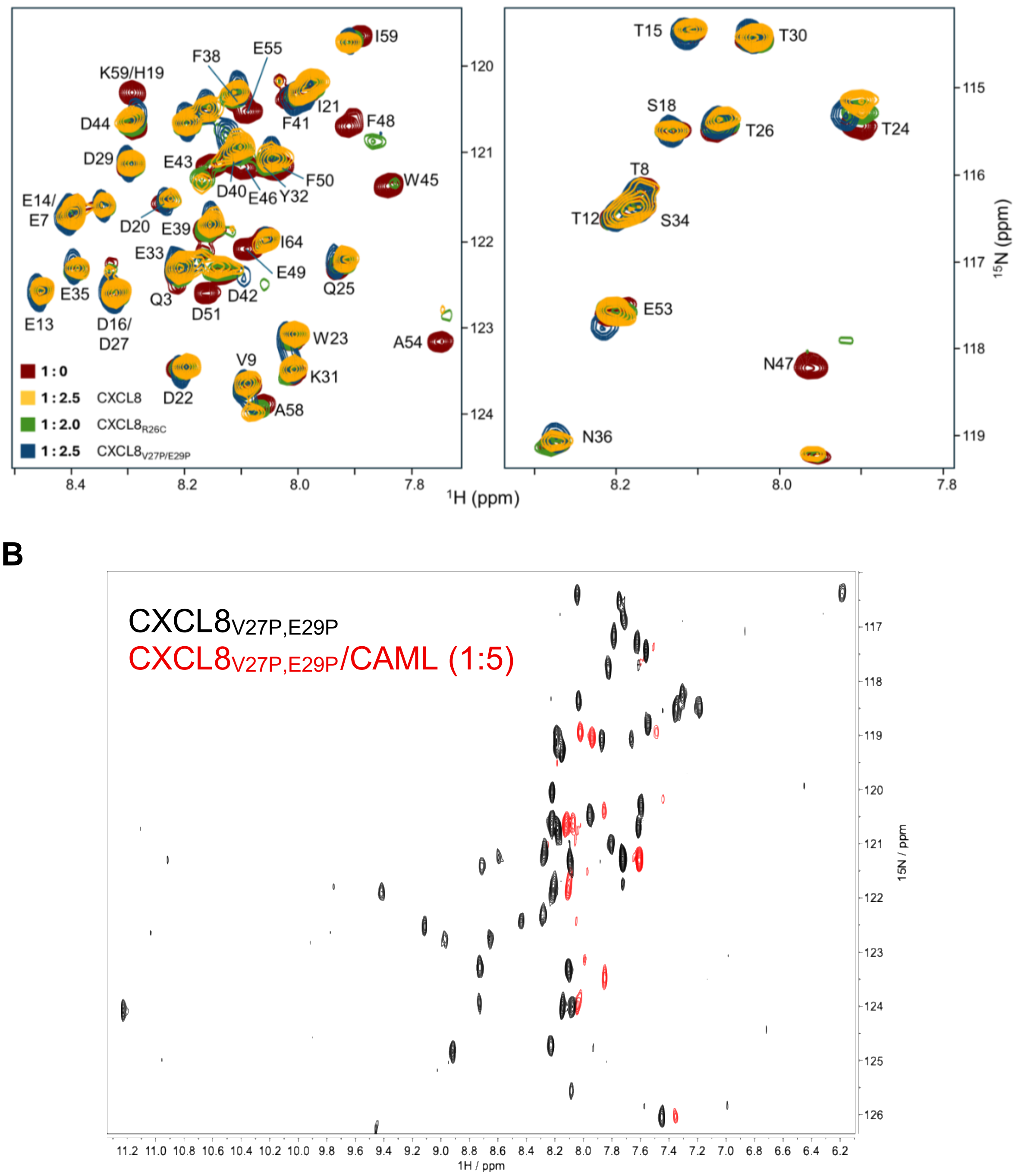

Fig. S9 NMR titrations of CAML$_{CT\text{-}NT}$ -CXCL8 interaction with monomer and dimer mutants A) Regions of the ^{1}H-^{15}N HSQC NMR spectra for CAML showing binding-induced chemical shift changes for trapped wild-type CXCL8, CXCL8$_{V27P/E29P}$ monomer mutant and CXCL8$_{R36C}$ dimer mutant. Highly similar chemical shift perturbations confirm the same mode of interaction. B) Shift (in ^{1}H) overlay of NMR spectra from the reverse titration with ^{15}N labelled CXCL8$_{V27P/E29P}$ monomer mutant and CAML$_{CT\text{-}NT}$ showing the same line broadening effect occurs for the monomer as the wild type. The unbound CXCL8$_{V27P/E29P}$ peaks are in black, and the CAML-bound peaks are in red.

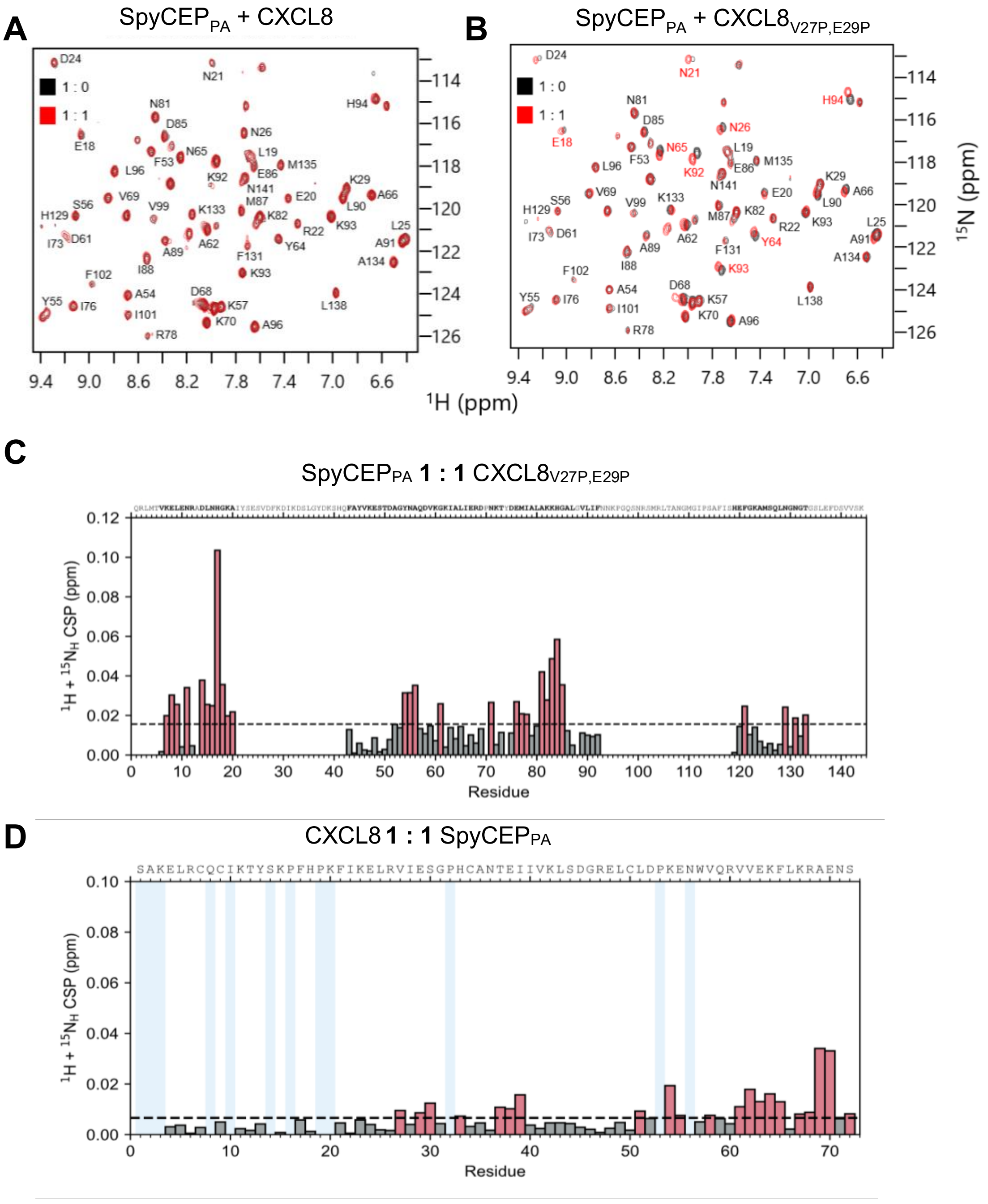

Figure S10 Mapping the SpyCEP$_{PA}$ domain-CXCL8 binding interfaces A) Region from the ^{1}H-^{15}N HSQC NMR spectra for SpyCEP$_{PA}$ showing small CXCL8 binding-induced chemical shift changes. The unbound peaks are in black, and the final bound peaks are in purple B) Bar charts of chemical shift perturbations for ^{1}H-^{15}N HSQC spectra of SpyCEP$_{PA}$ domain in the presence of 1:1 monomeric CXCL8$_{V27P,E29P}$. C) Bar charts of chemical shift perturbations for ^{1}H-^{15}N HSQC spectra of WT CXCL8 in the presence of 1:5 SpyCEP$_{PA}$ domain. Standard deviations are indicated as a dotted lines. Blue shaded bars represent peaks that could not be tracked through the titration.

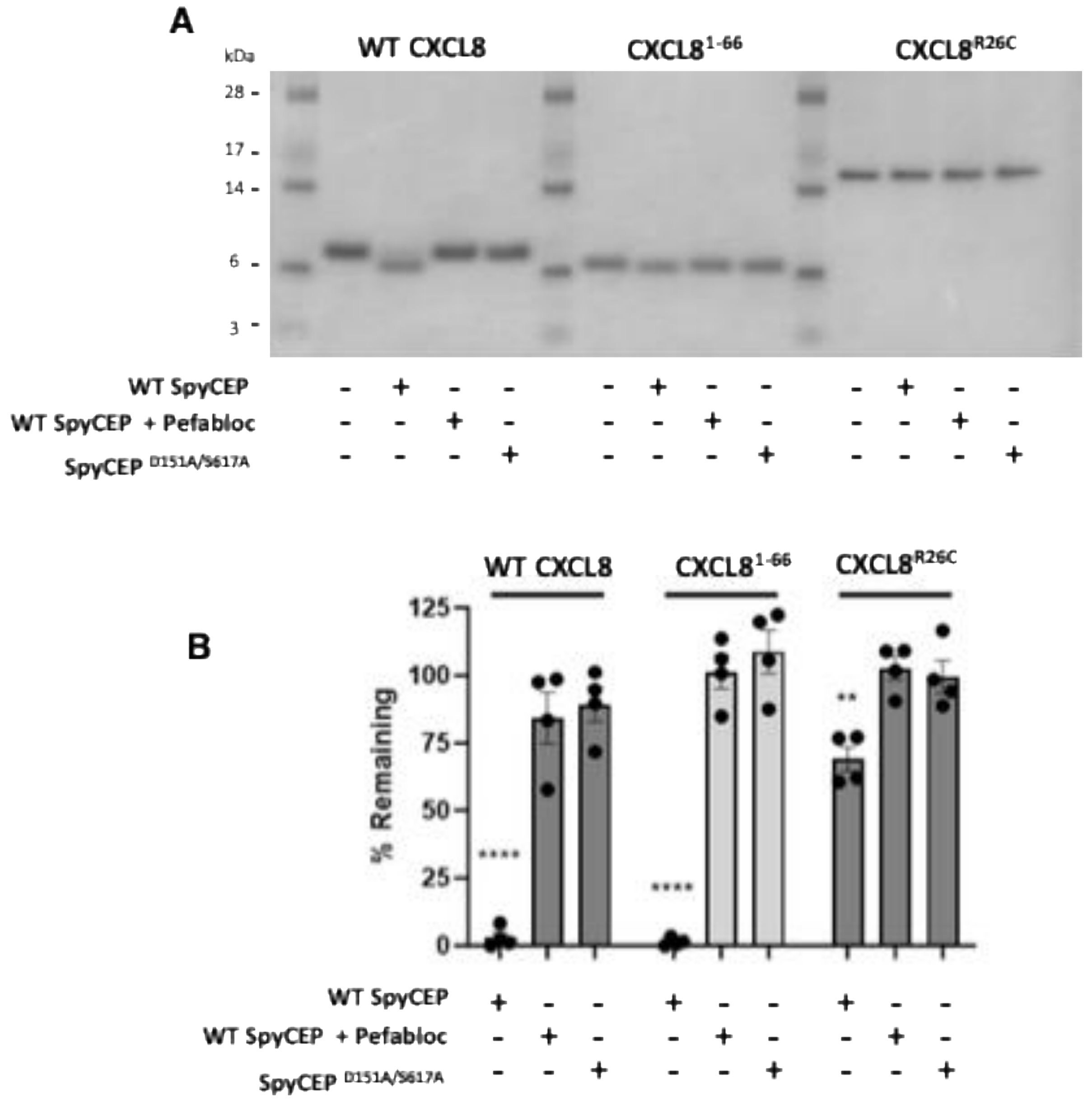

Fig. S11. **SpyCEP cleavage assays with obligate SpyCEP dimer and monomer mutants** (A) Chemokines were incubated for 16hr at 37C in the presence of either WT SpyCEP, WT SpyCEP in the presence of 2mg/mL Pefabloc or inactive SpyCEP variant. Digests were resolved by reducing SDS-PAGE and are representative of 3 separate experiments. (B) Cleavage of CXCL8 variants (dimer – $CXCL8_{R26C}$ and monomer – $CXCL8_{1\text{-}66}$) in identical digests as described in panel A was quantified using a CXCL8 ELISA and is shown as a percentage of the original substrate concentration, prior to digestion. Data are displayed as mean ± SEM of four separate experiments. Statistical analysis was performed via 2-way ANOVA, with multiple comparisons, followed by Dunnett's post-test.

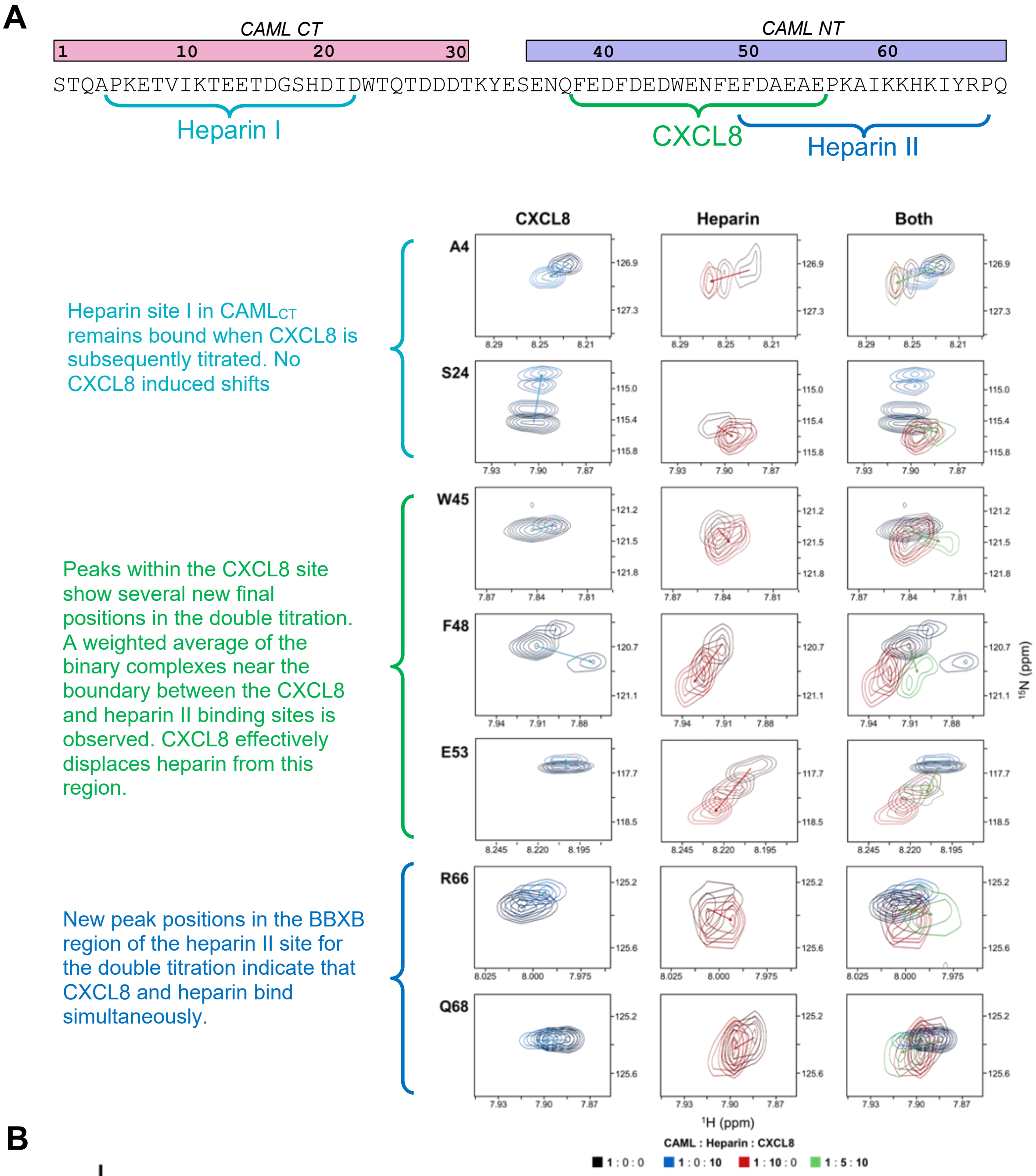

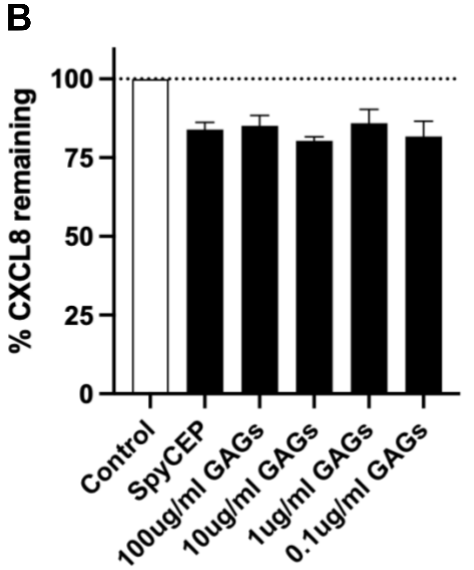

Fig. S12 Heparin and CXCL8 binding surfaces on CAML are mutually inclusive. A) Top: SpyCEP CAML sequence showing approximate CXCL8/heparin binding regions. Bottom: Regions of the ^{1}H-^{15}N HSQC NMR spectra for CAML showing heparin and CXCL8 binding-induced chemical shift changes. B) ELISA cleavage assay for SpyCEP (5 pmol CXCL8 at a 1:10 ratio) in the presence of heparan sulfate (0.1 µM-100 µM)

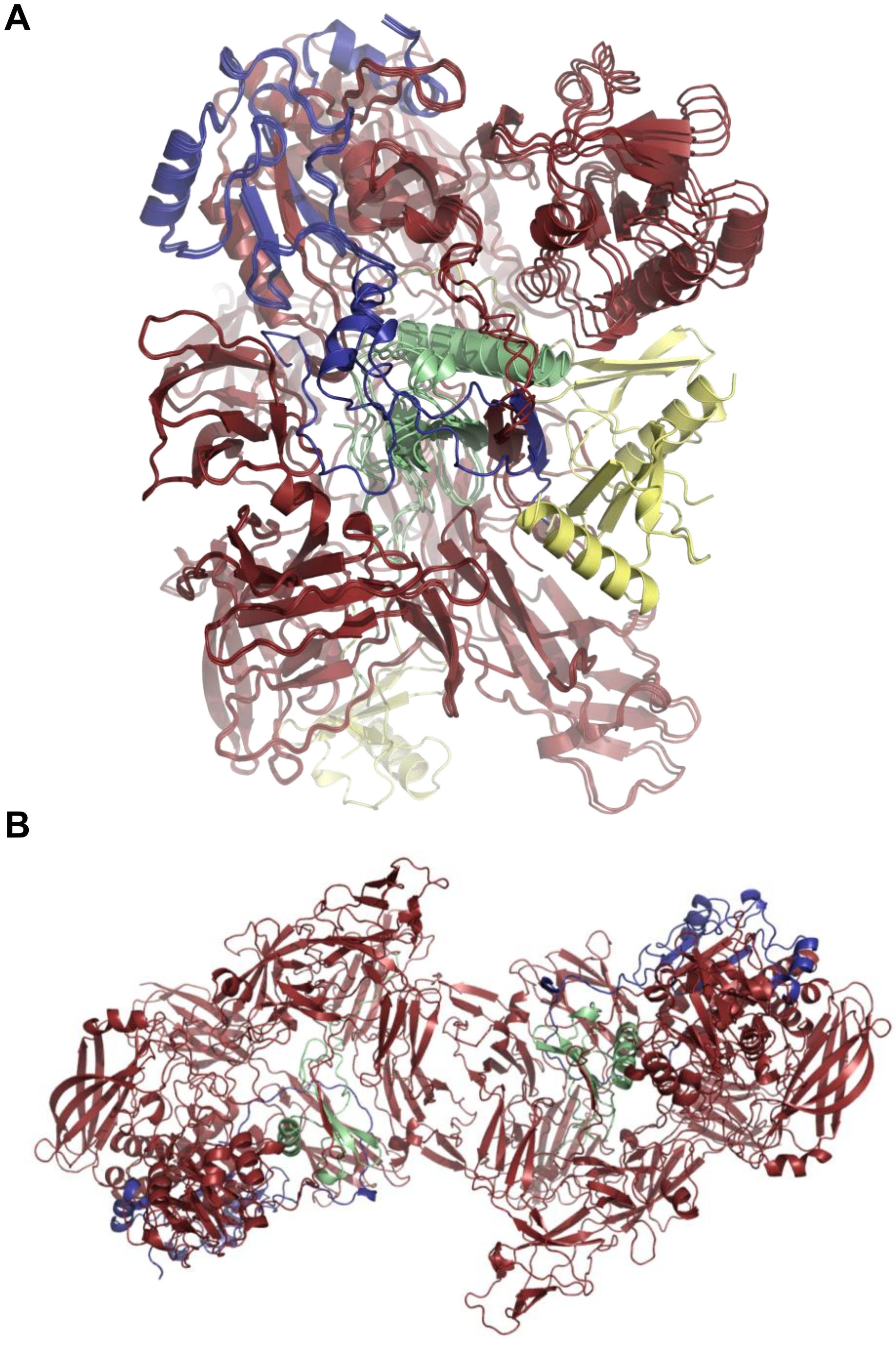

Fig. S13 AF3 Prediction of SpyCEP-CXCL8 dimer complexes. A) Representative top structures for one SpyCEP with two molecules of CXCL8. SpyCEP$_{NT}$ and SpyCEP$_{CT}$ shown in blue and red cartoons, while bound CXCL8 protomer 1 and unbound protomer 2 shown in green and yellow cartoons respectively. No inconsistent location for the second CXCL8, whereas the first is bound in the expected manner B) Two molecules of both SpyCEP and CXCL8. SpyCEP$_{NT}$ and SpyCEP$_{CT}$ shown in blue and red cartoons while bound CXCL8 protomers shown in green. Top model shown for clarity.

A

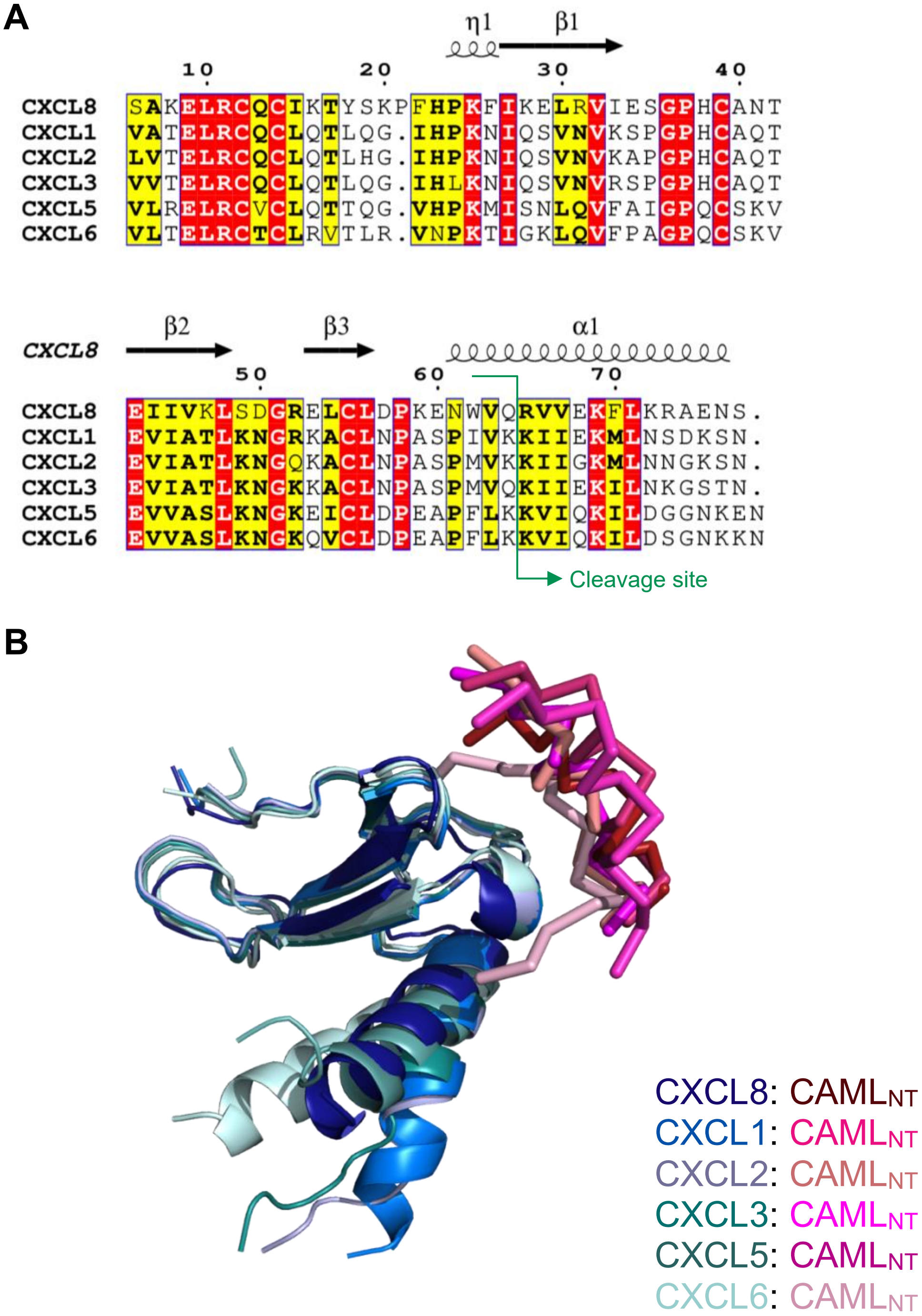

Fig. S14 ELR+CXC chemokines are cleaved by SpyCEP A) Sequence alignment for ELR+CXC chemokines that have been shown to be cleaved by SpyCEP (CXCL1, CXCL2, CXCL3, CXCL5, CXCL6 and CXCL8). Proposed conserved cleavage site shown in green. B) Superposition of top Alphafold3 structure predictions for complexes of full-length SpyCEP with CXCL1, CXCL2, CXCL3, CXCL5, CXCL6 and CXCL8. For clarity, only the acidic, aromatic-rich binding site in the CAML is shown for each SpyCEP structure.

Table S1 CryoEM collection, processing and refinement

	$SpyCEP_{DASA}$ + CXCL8	$SpyCEP_{DASA}$ × CXCL8	$SpyCEP_{DASA}$ + 3F2G10 PDB-31MR	$SpyCEP_{DASA}$ + 10B6C10
**Data collection and processing**				
Magnification	120,000	100,000	79,000	100,000
Voltage (kV)	300	200	200	200
Electron exposure ($e^{-}/Å^2$)	40	50	40	50
Defocus range (μm)	-1 to -2.5	-1 to -2.5	-1 to -2.5	-1 to -2.5
Pixel size (Å)	0.67	1.16	0.75 (2x EER of 1.5)	1.16
Symmetry imposed	C1	C1	C1	C1
Initial particle images (no.)	1,105,137	739,001	931,062	223,716
Final particle images (no.)	96,737	34,558	660,420	44,200
Map resolution (Å)	3.61	6.35 (3D Class)	3.07	5.50
FSC threshold	0.143	0.143	0.143	0.143
Map resolution range (Å)	6.33 to 3.16	8.05 to 5.61 (from NU Refinement of 3D Class)	4.09 to 2.78	7.29 to 3.70
cFAR	0.122	0.429	0.172	0.207
SCF	0.802	0.952	0.609	0.944
**Refinement**				
Initial model used (PBD code)	$SpyCEP_{DASA}$ + 3F2G10	-	Alphafold prediction	-
Model resolution (Å)	3.07	-	-	-
FSC threshold	0.143	-	-	-
Map sharpening B factor ($Å^2$)	-139.0	-497.9	-129.9	-102.9
Model composition				
Non-hydrogen atoms	10,547	-	12,670	-
Protein residues	1356	-	1399 (SpyCEP), 122 (VH), 113 (VL)	-
Ligands	0	-	0	-
R.M.S. deviations				

Bond lengths (Å)	xxx	-	xxx	-
Bond angles (°)	xxx	-	xxx	-
Validation				
MolProbity score	1.18	-	1.38	-
Clashscore	1.19	-	2.07	-
Poor rotamers (%)	0	-	0	-
Ramachandran plot				
Favoured (%)	94.97	-	94.03	-
Allowed (%)	5.03	-	5.97	-
Disallowed (%)	0	-	0	-
Ramachandran Z-score	-0.42 ± 0.22	-	-1.14 ± 0.20	-
Q-score	0.51	-	0.58	-

Table S2 Native Mass spectrometry measurements

Protein	Expected mass	Measured mass
CXCL8	8,381	8,380 ± 1<br>*9,602 ± 1
CXCL8 dimer	17,974	17,984 ±1
SpyCEP$_{NT}$ D151A	24,574	
SpyCEP$_{CT}$ S617A	151,860	151,725 ± 8
SpyCEP$_{DASA}$	176,417	177,886 ± 48<br>177,676 ± 8<br>177,427 ± 21
SpyCEP$_{DASA}$ dimer	352,834	347,052 ± 54
SpyCEP$_{DASA}$: CXCL8 (1:1)	184,780	185,955 ± 20

*9602 species is trace amounts of additional cleaved product of the CXCL8 fusion protein. Also note that SpyCEP is expected to bind calcium ions, but these are not included in the expected mass calculations.

Table S3 Glycan probes included in the focused GAG oligosaccharide microarray.

No	Probe	Sequence
1	HA-S6-DH	GlcAβ-3GlcNAcβ-4GlcAβ-3GlcNAcβ-4GlcAβ-3GlcNAc-DH
2	HA-S10-DH	GlcAβ-3GlcNAcβ-4GlcAβ-3GlcNAcβ-4GlcAβ-3GlcNAcβ-4GlcAβ-3GlcNAcβ-4GlcAβ-3GlcNAc-DH
3	HA-S14-DH	GlcAβ-3GlcNAcβ-4GlcAβ-3GlcNAcβ-4GlcAβ-3GlcNAcβ-4GlcAβ-3GlcNAcβ-4GlcAβ-3GlcNAcβ-4GlcAβ-3GlcNAcβ-4GlcAβ-3GlcNAc-DH
4	HA-S18-DH	GlcAβ-3GlcNAcβ-4GlcAβ-3GlcNAcβ-4GlcAβ-3GlcNAcβ-4GlcAβ-3GlcNAcβ-4GlcAβ-3GlcNAcβ-4GlcAβ-3GlcNAcβ-4GlcAβ-3GlcNAcβ-4GlcAβ-<br>3GlcNAcβ-4GlcAβ-3GlcNAc-DH
5	CSA-6-AO	ΔUA-3GalNAcβ-4GlcAα-3GalNAcβ-4GlcAα-3GalNAc-AO<br>SU-4 SU-4 SU-4
6	CSA-10-AO	ΔUA-3GalNAcβ-4GlcAβ-3GalNAcβ-4GlcAβ-3GalNAcβ-4GlcAβ-3GalNAcβ-4GlcAβ-3GalNAc-AO<br>SU-4 SU-4 SU-4 SU-4 SU-4
7	CSA-14-AO	ΔUA-3GalNAcβ-4GlcAβ-3GalNAcβ-4GlcAβ-3GalNAcβ-4GlcAβ-3GalNAcβ-4GlcAβ-3GalNAcβ-4GlcAβ-3GalNAcβ-4GlcAβ-3GalNAc-AO<br>SU-4 SU-4 SU-4 SU-4 SU-4 SU-4 SU-4
8	CSA-18-AO	ΔUA-3GalNAcβ-4GlcAβ-3GalNAcβ-4GlcAβ-3GalNAcβ-4GlcAβ-3GalNAcβ-4GlcAβ-3GalNAcβ-4GlcAβ-3GalNAcβ-4GlcAβ-3GalNAcβ-4GlcAβ-3GalNAcβ-4GlcAβ-3GalNAcβ-4GlcAβ-3GalNAc-AO<br>SU-4 SU-4 SU-4 SU-4 SU-4 SU-4 SU-4 SU-4 SU-4
9	CSB-6-AO	ΔUA-3GalNAcβ-4IdoAα-3GalNAcβ-4IdoAα-3GalNAc-AO<br>SU-4 SU-4 SU-4
10	CSB-10-AO	ΔUA-3GalNAcβ-4IdoAα-3GalNAcβ-4IdoAα-3GalNAcβ-4IdoAα-3GalNAcβ-4IdoAα-3GalNAc-AO<br>SU-4 SU-4 SU-4 SU-4 SU-4
11	CSB-14-AO	ΔUA-3GalNAcβ-4IdoAα-3GalNAcβ-4IdoAα-3GalNAcβ-4IdoAα-3GalNAcβ-4IdoAα-3GalNAcβ-4IdoAα-3GalNAcβ-4IdoAα-3GalNAc-AO<br>SU-4 SU-4 SU-4 SU-4 SU-4 SU-4 SU-4
12	CSB-18-AO	ΔUA-3GalNAcβ-4IdoAα-3GalNAcβ-4IdoAα-3GalNAcβ-4IdoAα-3GalNAcβ-4IdoAα-3GalNAcβ-4IdoAα-3GalNAcβ-4IdoAα-3GalNAcβ-4IdoAα-3GalNAcβ-4IdoAα-3GalNAc-AO<br>SU-4 SU-4 SU-4 SU-4 SU-4 SU-4 SU-4 SU-4 SU-4
13	CSC-6-AO	ΔUA-3GalNAcβ-4GlcAβ-3GalNAcβ-4GlcAβ-3GalNAc-AO<br>SU-6 SU-6 SU-6
14	CSC-10-AO	ΔUA-3GalNAcβ-4GlcAβ-3GalNAcβ-4GlcAβ-3GalNAcβ-4GlcAβ-3GalNAcβ-4GlcAβ-3GalNAc-AO<br>SU-6 SU-6 SU-6 SU-6 SU-6
15	CSC-14-AO	ΔUA-3GalNAcβ-4GlcAβ-3GalNAcβ-4GlcAβ-3GalNAcβ-4GlcAβ-3GalNAcβ-4GlcAβ-3GalNAcβ-4GlcAβ-3GalNAcβ-4GlcAβ-3GalNAc-AO<br>SU-6 SU-6 SU-6 SU-6 SU-6 SU-6 SU-6
16	CSC-18-AO	ΔUA-3GalNAcβ-4GlcAβ-3GalNAcβ-4GlcAβ-3GalNAcβ-4GlcAβ-3GalNAcβ-4GlcAβ-3GalNAcβ-4GlcAβ-3GalNAcβ-4GlcAβ-3GalNAcβ-4GlcAβ-3GalNAcβ-4GlcAβ-3GalNAcβ-4GlcAβ-3GalNAc-AO<br>SU-6 SU-6 SU-6 SU-6 SU-6 SU-6 SU-6 SU-6 SU-6
17	CSD-4-AO	SU-2<br>ΔUA-3GalNAcβ-4GlcAβ-3GalNAc-AO<br>SU-6 SU-2 SU-6
18	CSD-6-AO	SU-2<br>ΔUA-3GalNAcβ-4GlcAβ-3GalNAcβ-4GlcAβ-3GalNAc-AO<br>SU-6 SU-2 SU-6 SU-2 SU-6
19	CSD-8-AO	SU-2<br>ΔUA-3GalNAcβ-4GlcAβ-3GalNAcβ-4GlcAβ-3GalNAcβ-4GlcAβ-3GalNAc-AO<br>SU-6 SU-2 SU-6 SU-2 SU-6 SU-2 SU-6
20	CSD-10-AO	SU-2<br>ΔUA-3GalNAcβ-4GlcAβ-3GalNAcβ-4GlcAβ-3GalNAcβ-4GlcAβ-3GalNAcβ-4GlcAβ-3GalNAc-AO<br>SU-6 SU-2 SU-6 SU-2 SU-6 SU-2 SU-6 SU-2 SU-6
21	CSD-12-AO	SU-2<br>ΔUA-3GalNAcβ-4GlcAβ-3GalNAcβ-4GlcAβ-3GalNAcβ-4GlcAβ-3GalNAcβ-4GlcAβ-3GalNAcβ-4GlcAβ-3GalNAc-AO<br>SU-6 SU-2 SU-6 SU-2 SU-6 SU-2 SU-6 SU-2 SU-6 SU-2 SU-6
22	CSD-14-AO	SU-2<br>ΔUA-3GalNAcβ-4GlcAβ-3GalNAcβ-4GlcAβ-3GalNAcβ-4GlcAβ-3GalNAcβ-4GlcAβ-3GalNAcβ-4GlcAβ-3GalNAcβ-4GlcAβ-3GalNAc-AO<br>SU-6 SU-2 SU-6 SU-2 SU-6 SU-2 SU-6 SU-2 SU-6 SU-2 SU-6 SU-2 SU-6
23	Hep-4-AO	SU-2<br>ΔUA-4GlcNSα-4IdoAα-4GlcNS-AO<br>SU-6 SU-2 SU-6

No	Probe	Sequence
24	Hep-6-AO	SU-2<br>ΔUA-4GlcNSα-4IdoAα-4GlcNSα-4IdoAα-4GlcNS-AO<br>SU-6 SU-2 SU-6 SU-2 SU-6
25	Hep-8-AO	SU-2<br>ΔUA-4GlcNSα-4IdoAα-4GlcNSα-4IdoAα-4GlcNSα-4IdoAα-4GlcNS-AO<br>SU-6 SU-2 SU-6 SU-2 SU-6 SU-2 SU-6
26	Hep-10-AO	SU-2<br>ΔUA-4GlcNSα-4IdoAα-4GlcNSα-4IdoAα-4GlcNSα-4IdoAα-4GlcNSα-4IdoAα-4GlcNS-AO<br>SU-6 SU-2 SU-6 SU-2 SU-6 SU-2 SU-6 SU-2 SU-6
27	Hep-12-AO	SU-2<br>ΔUA-4GlcNSα-4IdoAα-4GlcNSα-4IdoAα-4GlcNSα-4IdoAα-4GlcNSα-4IdoAα-4GlcNSα-4IdoAα-4GlcNS-AO<br>SU-6 SU-2 SU-6 SU-2 SU-6 SU-2 SU-6 SU-2 SU-6 SU-2 SU-6
28	Hep-14-AO	SU-2<br>ΔUA-4GlcNSα-4IdoAα-4GlcNSα-4IdoAα-4GlcNSα-4IdoAα-4GlcNSα-4IdoAα-4GlcNSα-4IdoAα-4GlcNSα-4IdoAα-4GlcNS-AO<br>SU-6 SU-2 SU-6 SU-2 SU-6 SU-2 SU-6 SU-2 SU-6 SU-2 SU-6 SU-2 SU-6
29	Hep-18-AO	SU-2<br>ΔUA-4GlcNSα-4IdoAα-4GlcNSα-4IdoAα-4GlcNSα-4IdoAα-4GlcNSα-4IdoAα-4GlcNSα-4IdoAα-4GlcNSα-4IdoAα-4GlcNSα-4IdoAα-4GlcNSα-4IdoAα-4GlcNS-AO<br>SU-6 SU-2 SU-6 SU-2 SU-6 SU-2 SU-6 SU-2 SU-6 SU-2 SU-6 SU-2 SU-6 SU-2 SU-6 SU-2 SU-6
30	2-O-DeS Hep-8-AO	ΔUA-4GlcNSα-4IdoAα-4GlcNSα-4IdoAα-4GlcNSα-4IdoAα-4GlcNS-AO<br>SU-6 SU-6 SU-6 SU-6
31	2-O-DeS Hep-10-AO	ΔUA-4GlcNSα-4IdoAα-4GlcNSα-4IdoAα-4GlcNSα-4IdoAα-4GlcNSα-4IdoAα-4GlcNS-AO<br>SU-6 SU-6 SU-6 SU-6 SU-6
32	6-O-DeS Hep-8-AO	SU-2<br>ΔUA-4GlcNSα-4IdoAα-4GlcNSα-4IdoAα-4GlcNSα-4IdoAα-4GlcNS-AO<br>SU-2 SU-2 SU-2
33	6-O-DeS Hep-10-AO	SU-2<br>ΔUA-4GlcNSα-4IdoAα-4GlcNSα-4IdoAα-4GlcNSα-4IdoAα-4GlcNSα-4IdoAα-4GlcNS-AO<br>SU-2 SU-2 SU-2 SU-2
34	N-DeS Hep-8-AO	SU-2<br>ΔUA-4GlcNH2α-4IdoAα-4GlcNH2α-4IdoAα-4GlcNH2cα-4IdoAα-4GlcNH2-AO<br>SU-6 SU-2 SU-6 SU-2 SU-6 SU-2 SU-6
35	N-DeS Hep-10-AO	SU-2<br>ΔUA-4GlcNH2α-4IdoAα-4GlcNH2α-4IdoAα-4GlcNH2α-4IdoAα-4GlcNH2α-4IdoAα-4GlcNH2-AO<br>SU-6 SU-2 SU-6 SU-2 SU-6 SU-2 SU-6 SU-2 SU-6
36	N-DeS ReAc Hep-8-AO	SU-2<br>ΔUA-4GlcNAcα-4IdoAα-4GlcNAcα-4IdoAα-4GlcNAcα-4IdoAα-4GlcNAc-AO<br>SU-6 SU-2 SU-6 SU-2 SU-6 SU-2 SU-6
37	N-DeS ReAc Hep-10-AO	SU-2<br>ΔUA-4GlcNAcα-4IdoAα-4GlcNAcα-4IdoAα-4GlcNAcα-4IdoAα-4GlcNAcα-4IdoAα-4GlcNAc-AO<br>SU-6 SU-2 SU-6 SU-2 SU-6 SU-2 SU-6 SU-2 SU-6
38	N-DeS ReAc Hep-12-AO	SU-2<br>ΔUA-4GlcNAcα-4IdoAα-4GlcNAcα-4IdoAα-4GlcNAcα-4IdoAα-4GlcNAcα-4IdoAα-4GlcNAcα-4IdoAα-4GlcNAc-AO<br>SU-6 SU-2 SU-6 SU-2 SU-6 SU-2 SU-6 SU-2 SU-6 SU-2 SU-6
39	HS-S6-AO	GlcAβ-4GlcNAcα-4GlcAβ-4GlcNAcα-4GlcAβ-4aMan-AO<br>(May contain small amounts of GlcNS(6S) and IdoA(2S); some GlcNAc may be non-sulfated)
40	HS-S8-AO	GlcAβ-4GlcNAcα-4GlcAβ-4GlcNAcα-4GlcAβ-4GlcNAcα-4GlcAβ-4aMan-AO<br>(May contain small amounts of GlcNS(6S) and IdoA(2S); some GlcNAc may be non-sulfated)
41	HS(Lyl)-4-AO	SU-2<br>ΔUA-4GlcNAcα-4GlcAβ-4GlcNS-AO<br>(May contain GlcNS(6S) and IdoA(2S); some GlcNAc may be sulfated)
42	HS(Lyl)-6-AO	SU-2<br>ΔUA-4GlcNAcα-4GlcAβ-4GlcNAcα-4GlcAβ-4GlcNS-AO<br>(May contain GlcNS(6S) and IdoA(2S); some GlcNAc may be sulfated)
43	HS(Lyl)-8-AO	SU-2<br>ΔUA-4GlcNAcα-4GlcAβ-4GlcNAcα-4GlcAβ-4GlcNAcα-4GlcAβ-4GlcNS-AO<br>(May contain GlcNS(6S) and IdoA(2S); some GlcNAc may be sulfated)
44	HS(Lyl)-10-AO	SU-2<br>ΔUA-4GlcNAcα-4GlcAβ-4GlcNAcα-4GlcAβ-4GlcNAcα-4GlcAβ-4GlcNAcα-4GlcAβ-4GlcNS-AO<br>(May contain GlcNS(6S) and IdoA(2S); some GlcNAc may be sulfated)
No	Probe	Sequence

45	HS(LyI)-12-AO	SU-2<br>| ΔUA-4GlcNAcα-4GlcAβ-4GlcNAcα-4GlcAβ-4GlcNAcα-4GlcAβ-4GlcNAcα-4GlcAβ-4GlcNAcα-4GlcAβ-4GlcNS-AO<br>(May contain GlcNS(6S) and IdoA(2S); some GlcNAc may be sulfated)
46	HS(LyI)-14-AO	SU-2<br>| ΔUA-4GlcNAcα-4GlcAβ-4GlcNAcα-4GlcAβ-4GlcNAcα-4GlcAβ-4GlcNAcα-4GlcAβ-4GlcNAcα-4GlcAβ-4GlcNAcα-4GlcAβ-4GlcNS-AO<br>(May contain GlcNS(6S) and IdoA(2S); some GlcNAc may be sulfated)
47	HS(LyI)-16-AO	SU-2<br>| ΔUA-4GlcNAcα-4GlcAβ-4GlcNAcα-4GlcAβ-4GlcNAcα-4GlcAβ-4GlcNAcα-4GlcAβ-4GlcNAcα-4GlcAβ-4GlcNAcα-4GlcAβ-4GlcNAcα-4GlcAβ-<br>4GlcNS-AO<br>(May contain GlcNS(6S) and IdoA(2S); some GlcNAc may be sulfated)
48	HS(LyI)-18-AO	SU-2<br>| ΔUA-4GlcNAcα-4GlcAβ-4GlcNAcα-4GlcAβ-4GlcNAcα-4GlcAβ-4GlcNAcα-4GlcAβ-4GlcNAcα-4GlcAβ-4GlcNAcα-4GlcAβ-4GlcNAcα-4GlcAβ-<br>4GlcNAcα-4GlcAβ-4GlcNS-AO<br>(May contain GlcNS(6S) and IdoA(2S); some GlcNAc may be sulfated)
49	HS(LyI)-20-AO	SU-2<br>| ΔUA-4GlcNAcα-4GlcAβ-4GlcNAcα-4GlcAβ-4GlcNAcα-4GlcAβ-4GlcNAcα-4GlcAβ-4GlcNAcα-4GlcAβ-4GlcNAcα-4GlcAβ-4GlcNAcα-4GlcAβ-<br>4GlcNAcα-4GlcAβ-4GlcNAcα-4GlcAβ-4GlcNS-AO<br>(May contain GlcNS(6S) and IdoA(2S); some GlcNAc may be sulfated)
50	HS(LyIII)-6-AO	ΔUA-4GlcNAcα-4GlcAβ-4GlcNAcα-4GlcAβ-4GlcNAc-AO<br>(May contain GlcNS(6S) and IdoA(2S); some GlcNAc may be sulfated; reducing end GlcNS or GlcNAc)
51	HS(LyIII)-8-AO	ΔUA-4GlcNAcα-4GlcAβ-4GlcNAcα-4GlcAβ-4GlcNAcα-4GlcAβ-4GlcNAc-AO<br>(May contain GlcNS(6S) and IdoA(2S); some GlcNAc may be sulfated; reducing end GlcNS or GlcNAc)
52	HS(LyIII)-10-AO	ΔUA-4GlcNAcα-4GlcAβ-4GlcNAcα-4GlcAβ-4GlcNAcα-4GlcAβ-4GlcNAcα-4GlcAβ-4GlcNAc-AO<br>(May contain GlcNS(6S) and IdoA(2S); some GlcNAc may be sulfated; reducing end GlcNS or GlcNAc)
53	HS(LyIII)-12-AO	ΔUA-4GlcNAcα-4GlcAβ-4GlcNAcα-4GlcAβ-4GlcNAcα-4GlcAβ-4GlcNAcα-4GlcAβ-4GlcNAcα-4GlcAβ-4GlcNAc-AO<br>(May contain GlcNS(6S) and IdoA(2S); some GlcNAc may be sulfated; reducing end GlcNS or GlcNAc)
54	HS(LyIII)-14-AO	ΔUA-4GlcNAcα-4GlcAβ-4GlcNAcα-4GlcAβ-4GlcNAcα-4GlcAβ-4GlcNAcα-4GlcAβ-4GlcNAcα-4GlcAβ-4GlcNAcα-4GlcAβ-4GlcNAc-AO<br>(May contain GlcNS(6S) and IdoA(2S); some GlcNAc may be sulfated; reducing end GlcNS or GlcNAc)
55	HS(LyIII)-16-AO	ΔUA-4GlcNAcα-4GlcAβ-4GlcNAcα-4GlcAβ-4GlcNAcα-4GlcAβ-4GlcNAcα-4GlcAβ-4GlcNAcα-4GlcAβ-4GlcNAcα-4GlcAβ-4GlcNAcα-4GlcAβ-<br>4GlcNAc-AO<br>(May contain GlcNS(6S) and IdoA(2S); some GlcNAc may be sulfated; reducing end GlcNS or GlcNAc)
56	HS(LyIII)-18-AO	ΔUA-4GlcNAcα-4GlcAβ-4GlcNAcα-4GlcAβ-4GlcNAcα-4GlcAβ-4GlcNAcα-4GlcAβ-4GlcNAcα-4GlcAβ-4GlcNAcα-4GlcAβ-4GlcNAcα-4GlcAβ-<br>4GlcNAcα-4GlcAβ-4GlcNAc-AO<br>(May contain GlcNS(6S) and IdoA(2S); some GlcNAc may be sulfated; reducing end GlcNS or GlcNAc)
57	HS(LyIII)-20-AO	ΔUA-4GlcNAcα-4GlcAβ-4GlcNAcα-4GlcAβ-4GlcNAcα-4GlcAβ-4GlcNAcα-4GlcAβ-4GlcNAcα-4GlcAβ-4GlcNAcα-4GlcAβ-4GlcNAcα-4GlcAβ-<br>4GlcNAcα-4GlcAβ-4GlcNAcα-4GlcAβ-4GlcNAc-AO<br>(May contain GlcNS(6S) and IdoA(2S); some GlcNAc may be sulfated; reducing end GlcNS or GlcNAc)
58	KS-6-AO	Galβ-4GlcNAcβ-3Galβ-4GlcNAcβ-3Galβ-4GlcNAc(6S)-AO<br>(Variously 6S on Gal and GlcNAc)
59	KS-10-AO	Galβ-4GlcNAcβ-3Galβ-4GlcNAcβ-3Galβ-4GlcNAcβ-3Galβ-4GlcNAcβ-3Galβ-4GlcNAc(6S)-AO<br>(Variously 6S on Gal and GlcNAc)
60	KS-14-AO	Galβ-4GlcNAcβ-3Galβ-4GlcNAcβ-3Galβ-4GlcNAcβ-3Galβ-4GlcNAcβ-3Galβ-4GlcNAcβ-3Galβ-4GlcNAcβ-3Galβ-4GlcNAc(6S)-AO<br>(Variously 6S on Gal and GlcNAc)
61	KS-18-AO	Galβ-4GlcNAcβ-3Galβ-4GlcNAcβ-3Galβ-4GlcNAcβ-3Galβ-4GlcNAcβ-3Galβ-4GlcNAcβ-3Galβ-4GlcNAcβ-3Galβ-4GlcNAcβ-3Galβ-4GlcNAcβ-<br>3Galβ-4GlcNAc(6S)-AO<br>(Variously 6S on Gal and GlcNAc)

Table S4 Sample conditions of cryo-grid preparations.

Sample (3 µL)	Buffer	Grid	Concentration (µM)	Plasma clean time	Wait time	Blot time	Blot force	Humidity	Temperature
SpyCEP DASA + CXCL8 WT	150 mM NaCl, 40 mM tris, pH 7.5	Cu Quantifoil R2/2 (300)	5.35	90 sec	60 sec	6 sec	-2	100 %	4 ºC
SpyCEP DASA + CXCL8 WT + FA (2%)	150 mM NaCl, 20 mM Tris, pH 7.4	Au Quantifoil R1.2/1.3 (300)	1.94 (double application of 0.92)	90 sec	30 sec 30 sec	6 sec, 2 x 8 sec	-2	100 %	21 ºC
SpyCEP DASA + 3F2/G10	200 mM NaCl, 20 mM Tris, pH 8.0	Au Quantifoil R1.2/1.3 (300)	5.0	90 sec	30 sec	2 x 5 sec	-2	100 %	21 ºC
SpyCEP DASA + 10B6/C10	200 mM NaCl, 20 mM Tris, pH 7.0, 0.003% DDM	Au Quantifoil R1.2/1.3 (300)	5.0	90 sec	30 sec	2 x 6 sec	-2	100 %	21 ºC

Table S5 Supplemental glycan microarray document based on MIRAGE Guidelines (doi:10.3762/mirage.3)

<table>
<tr><th>Classification</th><th>Guidelines</th></tr>
<tr><td colspan="2">1. Sample: Glycan Binding Sample</td></tr>
<tr><td>Description of Sample</td><td><u>Sample names</u>: SpyCEP full-length, SpyCEP CAML$_{CT}$-NT, SUMO-CXCL8. All samples contained a N-terminal Hexa-His tag.
<u>Origin</u>: recombinant
<u>Method of preparation:</u> Please see the Materials and Methods in the main text.</td></tr>
<tr><td>Sample modifications</td><td>Not relevant.</td></tr>
<tr><td>Assay protocol</td><td>Microarray analyses were performed essentially as described (7), for modifications of the protocol please see "Glycan Microarray Analysis" under Materials and Methods.</td></tr>
<tr><td colspan="2">2. Glycan Library</td></tr>
<tr><td>Glycan description</td><td>The microarray (in house designation 'GAG oligosaccharide Array Set 12, 12b') contained 61 lipid-linked oligosaccharide probes, neoglycolipids (NGLs), derived from GAG oligosaccharides of HA, CSA, CSB, CSC, Heparin, HS and KS were prepared by. partial depolymerization of the polysaccharides as referenced below.
<table>
<tr><td>Poly-saccharides</td><td>Origins/sources</td><td>Reagents used for preparing oligosaccharide fractions</td><td>References</td></tr>
<tr><td>HA</td><td>Bovine vitreous humor (Sigma H7630)</td><td>Hyaluronidase (EC 3.2.1.35; from bovine testes; Sigma)</td><td>(8)</td></tr>
<tr><td>CSA</td><td>Bovine trachea Sigma C8529)</td><td rowspan="3">Chondroitin lyase ABC from Proteus vulgaris (EC4.2.2.4 ; Sigma)</td><td rowspan="3">(9)</td></tr>
<tr><td>CSB</td><td>Bovine mucosa (Sigma C3788)</td></tr>
<tr><td>CSC</td><td>Shark cartilage (Sigma C4384)</td></tr>
<tr><td>Heparin</td><td>Porcine intestinal mucosa (Sigma H3149)</td><td>Heparin lyase I (EC 4.2.2.7; Sigma)</td><td>(10)</td></tr>
<tr><td>HS</td><td>Porcine intestinal mucosa, Fraction I (Celsus Laboratories HO-10595)</td><td>Heparin Lyase III (E.C. 4.2.2.8; IBEX Technologies)</td><td>(11)</td></tr>
<tr><td>KS</td><td>Bovine corneal; from Robert Linhardt Lab.</td><td>Recombinant Keratanase II from Bacillus circulans (EC 3.2.1), from Robert Linhardt Lab.</td><td>(12)</td></tr>
</table>
The GAG oligosaccharide mixtures were size-fractionated by gel filtration (Bio-Gel P-6) chromatography, and analysed by negative-ion electrospray ionization mass spectrometry after conversion into their ammonium salts for determination of the chain lengths of the major components in the fractions (10).
Additional GAG oligosaccharides of CSD, HS (Hep Lyase I and Lyase III digested), and desulphated Hep were from a commercial supplier, Iduron,
The names and sequences of the 61 GAG NGL probes are in Supplementary Dataset 1.</td></tr>
</table>

Glycan modifications	With the exception of HA NGLs which were prepared from reducing HA oligosaccharides by reductive amination with the amino lipid, 1,2-dihexadecyl-*sn*-glycero-3-phosphoethanolamine (DHPE) (13); the rest GAG NGLs were prepared by oxime ligation with aminooxy functionalized DHPE (AOPE) (14).The details of the conditions used for GAG NGL preparation are as described (12).
**3. Printing Surface; e.g., Microarray Slide**	
Description of surface	Nitrocellulose-coated glass microarray slides.
Manufacturer	16-pad UniSart® 3D Microarray Slide from Sartorius (Goettingen, Germany)
Custom preparation of surface	Not relevant.
Non-covalent Immobilization	NGLs were formulated as liposomes by adding carrier lipids, 1,2-dihexanoyl-sn-glycero-3-phosphocholine (DHPC) and cholesterol, for robotically arraying and non-covalent immobilization on nitrocellulose-coated glass slides (7).
**4. Arrayer (Printer)**	
Description of Arrayer	Nano-Plotter 2.1 (GeSim, Radeberg, Germany)
Dispensing mechanism	Non-contact liquid delivery with four dispensing tips.
Glycan deposition	Approximately 0.33 nl was printed per spot.<br>NGL probes were printed at 2 and 5 fmol per spot in duplicate.
Printing conditions	The printing solutions were aqueous-based. Printing was performed at ambient temperature and relative humidity of 50-54%.<br>The printing solutions contained 100 pmol/µl each of cholesterol and DHPC as lipid carriers in addition to the lipid-linked glycan probes in water (HPLC grade). The concentrations of the lipid-linked glycan probes were 5 and 15 pmol/µl for the 2 and 5 fmol per spot levels, respectively.<br>All printing solutions contained Cy3 NHS ester (GE Healthcare) at 20 ng/ml (26 fmol/µl) as a marker to monitor the printing process.
**5. Glycan Microarray with "Map"**	
Array layout	Each array slide contained 16 subarrays (pads). Each pad was set up for printing 64 probes maximum, each at 2 levels in duplicate (four spots for one probe in a row); up to 256 spots (16x16) in total in each pad.
Glycan identification and quality control	The 61 glycan probes printed in 'GAG oligosaccharide Array Sets 12,12b' are defined in **Supplemental Dataset 1**. All the probes were well printed as shown by the Cy3 images of the printed slides. For quality control, the focused GAG arrays were analysed with various GAG-binding proteins and antibodies, as well as number of viruses and virial proteins. Predicted binding data were recorded. These data will be

	published and shared via the International Glycan Array Repository (https://glygen.ccrc.uga.edu/array/), which has entered its final testing phase.
**6. Detector and Data Processing**	
Scanning hardware	GenePix 4300A (Molecular Devices, Berkshire, UK)
Scanner settings	Scanning resolution: 10 um / pixel (this resolution is adequate for the sizes of sample spots)<br>Laser channel: Red (scan wavelength 635 nm)<br>PMT voltage: 350<br>Scan power: Adjusted for each sample to achieve maximum signal without saturation of any single spot. The laser powers used are indicated in **Supplementary Dataset 1.**
Image analysis software	GenePix® Pro 7 (Molecular Devices, Berkshire, UK) was used for quantitation of the fluorescent signal intensities from the array images.
Data processing	The gpr files were entered into an in-house microarray database. No particular normalization method, or statistical analysis was used.
**7. Glycan Microarray Data Presentation**	
Data presentation	The microarray binding results (fluorescence intensities and errors) are in the **Supplementary Dataset 1**, as well as **Figures 5A**. Binding results are presented as fluorescence intensity of binding in scores and errors.
**8. Interpretation and Conclusion from Microarray Data**	
Data interpretation	No software or algorithms were used to interpret processed data.
Conclusions	SpyCEP full-length and SpyCEP $CAML_{CT-NT}$ exhibited selective binding to heparin and HS probes, whereas SUMO-CXCL8 demonstrated robust, broad-spectrum binding across diverse sulphated GAG sequences, with the strongest overall binding signals.

Table S6 Enzyme mechanism pathway diagram for SpyCEP. Initial rates, rapid-equilibrium and quasi–steady-state approximations were used.

PROPOSED REACTION MECHANISM

Enzyme (E) = SpyCEP

Substrate (S)= CXCL8

S dimer (S_2)= $CXCL8_2$

Cofactor (C) = GAGs

Product (S*) = CXCL8*

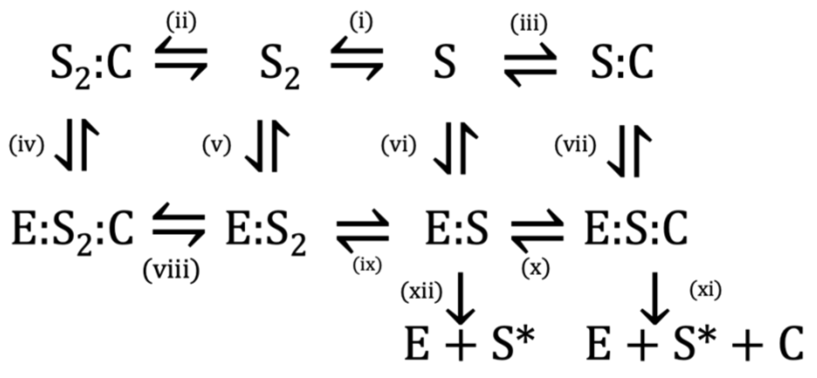

Step	Reaction	Estimates (reference/comment)
(i)	$2\,S \rightleftharpoons S_2$	$K_d^{(i)} = \sim 10\ \mu\text{M}$ (15)
(ii)	$S_2 + C \rightleftharpoons S_2 \cdot C$	$K_d^{(ii)} = \sim 10\ \mu\text{M}$ (16,17)
(iii)	$S + C \rightleftharpoons S \cdot C$	$K_d^{(iii)} \geq 100\ \mu\text{M}$ (16,17)
(iv)	$E + S_2 \cdot C \rightleftharpoons E \cdot S_2 \cdot C$	$K_d^{(iv)} \leq \sim 500$ nM (assumed same as (v) or tighter)
(v)	$E + S_2 \rightleftharpoons E \cdot S_2$	$K_d^{(v)} = \sim 500$ nM (this study - NMR titration in Fig. S6A)
(vi)	$E + S \rightleftharpoons E \cdot S$	$K_d^{(vi)} = 54 - 82$ nM (18)
(vii)	$E + S \cdot C \rightleftharpoons E \cdot S \cdot C$	$K_d^{(vii)} = 54 - 82\ n\text{M}$ (18)
(viii)	$E \cdot S_2 + C \rightleftharpoons E \cdot S_2 \cdot C$	$K_d^{(viii)} \leq \sim 10\ \mu\text{M}$ (assume same as (ii) or tighter)
(ix)	$E \cdot S + S \rightleftharpoons E \cdot S_2$	(ON) $K_d^{(ix)} = \sim 1$ mM (E induces S dimer dissociation - this study)<br>(OFF) $K_d^{(ix)} = \sim 1 \mu\text{M}$
(x)	$E \cdot S + C \rightleftharpoons E \cdot S \cdot C$	$K_d^{(x)} \leq 100\ \mu\text{M}$ (assume same as (iii) or tighter)
(xi)	$E \cdot S \xrightarrow{k_{1,\text{cat}}} E + S^*$	$k_{1,\text{cat}} = 1.6\ \text{s}^{-1}$ (18)
(xii)	$E \cdot S \cdot C \xrightarrow{k_{2,\text{cat}}} E + S^*$	(ON) $k_{2,\text{cat}} = 1.6\ \text{s}^{-1}$ (Similar E activity with GAGs - this study Fig. S13B)<br>(OFF) $k_{2,\text{cat}} = 0.05\ \text{s}^{-1}$

PREDICTIONS

Scenarios	[S]/ [CXCL8]	[E]/ [SpyCEP]	$[C]_{\text{eff}}$ [GAGS]	SpyCEP features - **OFF**<br>$K_d^{(ix)} = 1\ \mu\text{M}$ (No E dissociation of S_2)<br>$k_{2,\text{cat}} = 0.05\ \text{s}^{-1}$ (GAGs inhibit)<br>Time to deplete [S] by 50% (t_{50})	SpyCEP features - **ON**<br>$K_d^{(ix)} = 1$ mM (E dissociation of S_2)<br>$k_{2,\text{cat}} = 1.6\ \text{s}^{-1}$ (E activity with GAGs)<br>Time to deplete [S] by 50% (t_{50})
Infection site	1 μM	1 μM	500 μM	> 5s	< 1 s
Nearby tissue	100 nM	1nM	500 μM	> 5 mins	< 1 min
Systemic	10 nM	100 pM	5 μM	~ 5 mins	~ 5 mins

SI References

1. N. Vaidehi, J. E. Pease, R. Horuk, Modeling small molecule-compound binding to G-protein-coupled receptors. *Methods Enzymol* **460**, 263-288 (2009).
2. G. Schoofs, A. Van Hout, T. D'Huys, D. Schols, T. Van Loy, A Flow Cytometry-based Assay to Identify Compounds That Disrupt Binding of Fluorescently-labeled CXC Chemokine Ligand 12 to CXC Chemokine Receptor 4. *J Vis Exp* 10.3791/57271 (2018).
3. P. Zengel *et al.*, μ-Slide Chemotaxis: a new chamber for long-term chemotaxis studies. *BMC Cell Biol* **12**, 21 (2011).
4. K. Wróblewski, M. Zalewski, A. Kuriata, S. Kmiecik, CABS-flex 3.0: an online tool for simulating protein structural flexibility and peptide modeling. *Nucleic Acids Research* **53**, W95-W101 (2025).
5. C. Dominguez, R. Boelens, A. M. J. J. Bonvin, HADDOCK: A Protein−Protein Docking Approach Based on Biochemical or Biophysical Information. *Journal of the American Chemical Society* **125**, 1731-1737 (2003).
6. K. M. Sepuru, K. Rajarathnam, Distinct Differences in Structural States of Conserved Histidines in Two Related Proteins: NMR Studies of the Chemokines CXCL1 and CXCL8 in the Free Form and Macromolecular Complexes. *Biochemistry* **57**, 5969-5977 (2018).
7. Y. Liu, R. A. Childs, A. S. Palma, M. A. Campanero-Rhodes, M. S. Stoll, Chai, W., T. Feizi, Neoglycolipid-based oligosaccharide microarray system: preparation of NGLs and their noncovalent immobilization on nitrocellulose-coated glass slides for microarray analyses. *Methods in molecular biology (Clifton, N.J.)* **808**, 117–136 (2012).
8. W. Chai, J. G. Beeson, H. Kogelberg, G. V. Brown, A. M. Lawson, Inhibition of adhesion of Plasmodium falciparum-infected erythrocytes by structurally defined hyaluronic acid dodecasaccharides. *Infection and immunity* **69**, 420–425 (2001).
9. W. Chai, J. G. Beeson, A. M. Lawson, A. M. The structural motif in chondroitin sulfate for adhesion of Plasmodium falciparum-infected erythrocytes comprises disaccharide units of 4-O-sulfated and non-sulfated N-acetylgalactosamine linked to glucuronic acid. *The Journal of Biological Chemistry 277*, 22438–22446 (2002).
10. W. Chai, J. Luo, C. K. Lim, A. M. Lawson, Characterization of heparin oligosaccharide mixtures as ammonium salts using electrospray mass spectrometry. *Analytical chemistry*, **70**, 2060–2066 (1998).
11. W. Chai, C. Leteux, C. Westling, U. Lindahl, T. Feizi, Relative susceptibilities of the glucosamine-glucuronic acid and N-acetylglucosamine-glucuronic acid linkages to heparin lyase III. *Biochemistry* **43**, 8590–8599 (2004).
12. N. Wu, L. M. Silva, Y. Liu, Y. Zhang, C. Gao, F. Zhang, L. Fu, Y. Peng, R. Linhardt, T. Kawasaki, B. Mulloy, W. Chai, T. Feizi, Glycan Markers of Human Stem Cells Assigned with Beam Search Arrays. *Molecular & cellular proteomics : MCP* **18**, 1981–2002 (2019).
13. W. Chai, M. S. Stoll, C. Galustian, A. M. Lawson, T. Feizi, Neoglycolipid technology: deciphering information content of glycome. *Methods in enzymology* **362**, 160–195 (2003).
14. Y. Liu, T. Feizi, M. A. Campanero-Rhodes, R. A. Childs, Y. Zhang, B. Mulloy, P. G. Evans, H. M. Osborn, D. Otto, P. R. Crocker, W. Chai, Neoglycolipid probes prepared via oxime ligation for microarray analysis of oligosaccharide-protein interactions. *Chemistry & biology* **14**, 847–859 (2007).
15. S. D. Burrows *et al.*, Determination of the monomer-dimer equilibrium of interleukin-8 reveals it is a monomer at physiological concentrations. *Biochemistry* **33**, 12741-12745 (1994).
16. P. R. Joseph, P. D. Mosier, U. R. Desai, K. Rajarathnam, Solution NMR characterization of chemokine CXCL8/IL-8 monomer and dimer binding to glycosaminoglycans: structural plasticity mediates differential binding interactions. *Biochem J* **472**, 121-133 (2015).

17. Mahler, B.P.; Nagarajan, B.; Sankaranarayanan, N.V.; Joseph, P.R.B.; Desai, U.R.; Rajarathnam, K. Structural Basis of Chemokine CXCL8 Monomer and Dimer Binding to Chondroitin Sulfate: Insights into Specificity and Plasticity. *Biomolecules* **16**, 124 (2026).
18. M. Pearson *et al.*, Structure-activity studies of Streptococcus pyogenes enzyme SpyCEP reveal high affinity for CXCL8 in the SpyCEP C-terminal. *Sci Rep* **13**, 19052 (2023).
